# Photocatalytic Water Splitting Promoted by 2D and 3D Porphyrin Covalent Organic Polymers Synthesized by Suzuki-Miyaura Carbon-Carbon Coupling

**DOI:** 10.3390/nano12183197

**Published:** 2022-09-14

**Authors:** Maria Novoa-Cid, Arianna Melillo, Belén Ferrer, Mercedes Alvaro, Herme G. Baldovi

**Affiliations:** 1 Department of Chemistry, Universitat Politècnica de València, 46022 Valencia, Spain; 2 Instituto de Tecnología Química CSIC-UPV, Universitat Politècnica de València, 46022 Valencia, Spain

**Keywords:** 5,10,15,20-Tetrakis-(4-bromophenyl)porphyrin, Suzuki–Miyaura coupling reaction, hydrogen generation, oxygen generation, solar fuels, photocatalytic water splitting, photocatalysis, covalent organic polymers, photoactive polymers, porphyrin, 2D, 3D

## Abstract

This work deals with the synthesis of metal-free and porphyrin-based covalent organic polymers (COPs) by the Suzuki–Miyaura coupling carbon-carbon bond forming reaction to study the photocatalytic overall water splitting performance. Apart from using 5,10,15,20-Tetrakis-(4-bromophenyl)porphyrin, we have chosen different cross-linker monomers to induce 2-dimensional (2D) or 3-dimensional (3D) and different rigidity in their resulting polymeric molecular structure. The synthesised COPs were extensively characterised to reveal that the dimensionality and flexibility of the molecular structure play an intense role in the physical, photochemical, and electronic properties of the polymers. Photoinduced excited state of the COPs was evaluated by nanosecond time-resolved laser transient absorption spectroscopy (TAS) by analysing excited state kinetics and quenching experiments, photocurrent density measurements and photocatalytic deposition of Ru^3+^ to RuO_2,_ and photocatalysis. In summary, TAS experiments demonstrated that the transient excited state of these polymers has two decay kinetics and exhibit strong interaction with water molecules. Moreover, photocurrent and photocatalytic deposition experiments proved that charges are photoinduced and are found across the COP molecular network, but more important charges can migrate from the surface of the COP to the medium. Among the various COPs tested, COP–3 that has a flexible and 3D molecular structure reached the best photocatalytic performances, achieving a photocatalytic yield of 0.4 mmol H_2_ × g_COP–3_^−1^ after 3 h irradiation.

## 1. Introduction

In the current ongoing shift from fossil fuels to renewable energies there is much interest in exploring the potential of using the excess electricity generated in converting water or CO_2_ into high value chemicals [1,2,3,4]. However, there is a direct pathway to produce chemicals from light, called photocatalysis, in which, sunlight energy is utilized to promote redox reaction [5,6]. The field of solar photocatalysis is dominated by the use of inorganic semiconductors, such as metal oxides as modified TiO_2_ or WO_3_ [7,8], chalcogenides [9,10], inorganic perovskites, and metallic oxo salts such as BiVO_4_ [11,12,13]. However, due to still unsatisfactory performance, the area is more recently expanding towards hybrid organic-inorganic materials such as metal organic frameworks (MOFs) [14,15,16]. In contrast to these photocatalysts exclusively containing an inorganic component or metal nodes in combination with an organic-based ligand, the development of purely organic materials as photocatalysts has been comparatively much less studied [17,18,19]. Recently, many authors have made mixtures of two semiconductors to overcome higher photocatalytic results with the formation of Z-scheme heterojunctions, achieving promising results in the field [20].

Covalent organic polymers (COPs) are porous polymer frameworks constituted by two different rigid monomers having two or more binding sites in specific directions, whose structure is defined by the alternated combination of the two monomers [21,22,23,24,25,26,27,28]. These materials share many similarities with covalent organic frameworks (COFs) in terms of high porosity and surface area, however, they have some notable differences. The main difference is that COFs are materials with a high degree of crystallinity due to their ordered molecular structure and defined pore diameter, whereas COPs are more amorphous, although they may have some crystalline domains in their structure; additionally, although they are also mesoporous, their pore diameter is more heterogeneous. Therefore, COPs have higher numbers of structural defects generating many active centers for catalysis. These solids are particularly appealing because of their chemical composition and their tunable surface chemistry [22,23,29]. Generally, one of the monomers has more than two binding sites that creates pores and an open accessible inner space [21,28]. As in the case of MOFs, materials based on reticulated polymer properties also depend on the properties of the constitutive monomers or linkers [26,30,31]. COP materials are under intense development due to their many applications in various fields, such as gas adsorption [32], biosensors [33], photocatalysis [6,34], electrocatalysis [35], and semiconductors in electronic devices [36]. Focusing on photocatalysis, optoelectronic and redox properties of these materials depend directly on the molecular properties of the monomers utilised in their fabrication. For instance, the introduction of polyaromatic molecules might increase electron mobility among the polymeric particles and photosensitizing dyes should increase light harvesting properties in COPs. Moreover, the introduction of metal complexes would enhance redox reactions [37].

In plants, porphyrins are key photosensitizer units for absorbing and transferring light energy to other parts of the plants in the photosynthetic system. They are considered an ideal model for constructing artificial photocatalysts. There is a vast number of materials in which porphyrins are used as photosensitizers to promote photocatalytic and electrocatalytic water splitting [38,39,40,41,42,43,44]. Several COFs and COPs use porphyrins to produce new photocatalysts. As an example, Osaka and co-workers reported a new strategy where 2D COFs constituted by the binding of an aldehyde derivative that reacts with 5,10,15,20-tetrakis(4-aminophenyl)-21H,23H-porphine) that in combination with Pt and rGO is able to generate hydrogen upon visible and near infrared light due to their light harvesting properties [33]. On the other hand, most of the published works have synthesized imine, boronic esters, or boroxine rings linked COPs and COFs, leading to poor stable polymers that could be hydrolysed in basic or acidic pH where the reaction of overall water splitting condition is favored [16,27]. Additionally, some authors have debated about the effect of dimensionality of the molecular framework in the photocatalyst properties in terms of catalytic efficiency and surface area [34]. For instance, 2D or 3D molecular structure of the framework had a direct impact on the total gas absorption capacity. In the present work, we prepare COPs via the Suzuki–Miyaura reaction coupling porphyrins with 2D and 3D linkers leading to COPs linked by carbon-carbon bonds, enhancing thermal and chemical stability. Recently, some authors have synthesized COPs and COFs following a similar synthetic procedure, but none of them have explored the feasibility of carbon-carbon bonded materials in photocatalysis or studied how photocatalytic efficiency is affected by the dimensionality or rigidity of the molecular network [45,46]. In addition to the 3D COPs, we also explored the photocatalytic performance depending on the flexibility of the resultant molecular network by choosing 3D linkers with different molecular rigidity. Our results show that dimensionality and rigidity of the polymeric networks play a prominent role in the final physical, photochemical, electrochemical, and photocatalytic activity.

## 2. Experimental Section

***Materials.*** 5,10,15,20-(Tetra-4-bromophenyl)porphyrin (Porph-Br4) with 98% purity) was purchased from Porphychem, Dijon, France. Pyrene-2,7-diboronic acid pinacol ester (96% purity) and tetra(4-hydroxyboryphenyl)methane (95% purity), both were acquired from ABCR. 2,2′,7,7′-Tetrakis(4,4,5,5-tetramethyl-1,3,2-dioxaborolan-2-yl)-9,9′-spirobi[fluorene] was synthesized using bis(pinacolato)diboron (99% purity), 2,2′,7,7′-tetrabromo-9,9′-spirobi[fluorene] (95% purity) (Porph-Br4), bis(triphenylphosphine)palladium(II) dichloride (≥99.0% purity), and potassium acetate (≥99.0% purity) from Merck (Sigma-Aldrich, San Luis, MO, USA). Sodium hydroxide (97% purity, powder), ammonium cerium (IV) nitrate (≥99.99% purity), ruthenium (III) chloride hydrate (99.98% purity), and lead (II) chloride (98% purity) were also acquired from Merck (Sigma-Aldrich, San Luis, MO, USA). Hydrochloric acid (37%, Pharmpur^®^, Königsbrunn, Alemania) was acquired from Scharlab.

***Characterization Techniques.*** Proton nuclear magnetic resonance (^1^H NMR) spectra were collected on a Bruker Advance 400 (400 MHz) spectrometer at 20 °C. Combustion chemical analysis was performed with a Fisons EA analyser. The amount of retained Pd in of the three COPs was measured with inductively coupled plasma optical emission spectroscopy (ICP-OES) with Perkin Elmer Optima 2100 DV instrument (Waltham, UK) analysis. N_2_ adsorption isotherms were measured at 77 K using a Micromeritics ASAP 2010 apparatus. Thermogravimetric analyses were performed on a TGA/SDTA851e METTLER TOLEDO station. The morphology and the composition of each material were characterized using a high-resolution field emission scanning electron microscope (HR-FESEM) model GeminiSEM 500 from Zeiss instruments, Oberkochen, Alemania; and equipped with Energy dispersive X-ray detector, EDS (OXFORD INSTRUMENTS, Abingdon, Reino Unido) for composition analysis. High resolution transmission electron microscopy images (HR-TEM) were recorded on a JEOL JEM 2100F instrument (Akishima, Japan) with an acceleration voltage of 200 kV coupled with a large area Energy-Dispersive X-ray spectroscopy detector (EDS detector), X-Max 80 of Oxford Instruments (Abingdon, UK). X-ray powder diffraction (XRD) patterns were acquired with a Bruker PANalytical Empyrean diffractometer (Malvern Instruments Limited, Malvern, UK) (Cu K_α_ radiation) in transmission geometry. Diffuse reflectance ultraviolet–visible–near infrared (UV–VIS–NIR) spectra were recorded on a Varian Cary 5000 spectrophotometer (Agilent Technologies, Santa Clara, CA, USA) having an integrating sphere and using BaSO_4_ as reference. H_2_ chemisorption experiments were carried out using the double isotherm method on a Quantachrome Autosorb-1C equipment. Prior to adsorption, the samples were pretreated in situ at 120 °C for 2 h (5 °C/min) on helium flow. Then, the temperature was lowered to 100 °C in He and finally, the samples were degassed at 1333 × 10^−3^ Pa for 2 h at 100 °C. Afterwards, the temperature was lowered at 40 °C. Then, pure H_2_ was administered and the first adsorption isotherm was measured. After evacuation at 40 °C, the second isotherm was measured. The amount of chemisorbed H_2_ was then obtained by subtracting the two isotherms. The pressure range studied was 0.5 to 11 × 10^4^ Pa.

Additional experimental data can be found in the Supporting Information.

## 3. Results and Discussion

In this section, the synthesis and characterization of the three COPs photocatalysts will be discussed first. Next, the photochemical characterization of the COPs will be presented by analysing their absorbance spectroscopy, photoluminescence, and nanosecond time-resolved transient absorption spectroscopy (TAS), in nanoseconds, which will provide evidence of the photoinduced charge separation behaviour. Then, the electrochemical properties of the materials will be described to conclude with an analysis of the photogenerated charges (e^−^/h^+^). Finally, in the last section, the photocatalytic activity of the polymers will be discussed, and a photocatalytic mechanism will be proposed.

### 3.1. Synthesis and Physical Characterization of COP Photocatalysts

**Figure 1:**
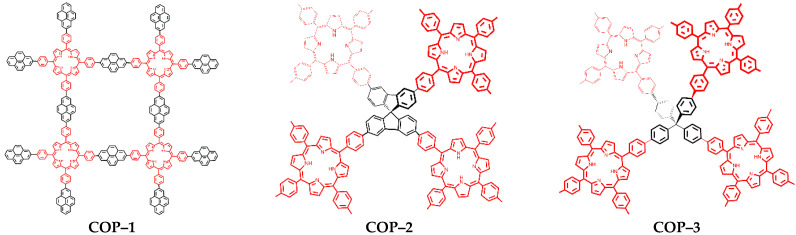
Simplified molecular structure of the three: COP–1, COP–2, and COP–3.

In the present study, three different COPs based on metal-free porphyrin were prepared having different cross-linker monomers with the aim of producing COPs with 2D or 3D molecular frameworks. COP–1 was prepared with a planar linker (2D) derived from pyrene which allowed π-bond delocalization double bond, whereas COP–2 and COP–3 had a tetrahedral linker (3D) spirobifluorene and tetraphenylmethane, respectively. Within 3D COPs, the reason to use different cross-linkers is to explore the effect of molecular rigidity in its physical and photochemical properties, as more molecular rigidity must translate in less degrees of freedom during the polymerization reaction, affecting the overall surface area, pore size, and physicochemical properties of the COP. The preparation route is illustrated in Scheme S1, while synthetic details can be found in the experimental section. One of the issues is the presence of residual amounts of the transition metals used as catalysts during the synthesis of the material. In the present case, the materials were prepared via Suzuki–Miyaura cross-coupling using organometallic Pd complex catalysts. It is not possible to avoid deposition of Pd nanoparticles (NPs) formed during the synthesis on the material, thus, the removal of Pd NPs requires an additional purification step. Among the various possible options to remove Pd, acid solutions (such an aqueous mixture (20% *v*/*v*) of 1:1 HNO_3_:HCl) were not desirable due to possible porphyrin protonation. The use of NaCN solution was considered to be more convenient since this treatment should not affect the porphyrin macro ring (Scheme S1c). However, elemental analysis reveals that after removing Pd NPs, we still detected the presence of 0.8% *w*/*w* of atomic Pd. Moreover, since ligands may not be completely consumed in the reaction, an additional washing step with Soxhlet is also necessary to remove reagents that remain adsorbed on the material (Scheme S1d). The structure of the three COPs synthesized is presented in Figure 1.

Combustion chemical analysis determines the percentage of carbon, hydrogen, and nitrogen of each of the three COPs. The corresponding values are provided in Table 1. The nitrogen percentage measured with this technique corresponds to the nitrogen present in the Porph-Br_4_ macro-rings and it is used to calculate the porphyrin content of the COPs. Moreover, the carbon percentage is used to calculate the cross-linker content for each COP. The analytical data with respect to the porphyrin/cross-linker molar ratio are included in Table S1. Considering that each boronated functional group of the cross-linker component reacts with each bromine atom of the porphyrin, the molar ratios of porphyrin/cross-linker should be 1:2, 1:1, and 1:1 for COP–1, COP–2, and COP–3, respectively. In the case of COP–2 and COP–3, the molar ratios match well with the expected stoichiometry used during synthesis; however, the molar ratio in the case of COP–1 is 1:1.8, which differs from 1:2, meaning that the porphyrin content is higher than expected.

Isothermal nitrogen adsorption measurements were performed to determine the specific surface area and porosity. A summary of the results is also included in Table 1. It can be seen that COP–2 and COP–3 having 3D cross-linker monomers exhibit a larger specific area than COP–1, in which both the porphyrin and the pyrene co-monomers are planar. In the case of 3D COPs, there is a great increase in surface area when the cross-linker used has less molecular rigidity. It is probable that in COP–3 during polymerization, molecules can separate more from each other due to steric impediment. However, the opposite trend is observed for the dimensions of the pores that are bigger for COP–1 compared with COP–2 and COP–3, probably due to the 3D property of the molecular structure. The decrease of the pore size could lead to a more active photocatalyst because we could start having the confinement effect phenomena inside the COP pores. In summary, the election of the cross-linker monomer has a direct impact on surface area and pore size; monomers with 3D molecular structure and flexibility will lead to polymers with larger surface area and smaller pore size.

FT-IR spectra of the three COPs are presented in Figure S2. As can be seen in that figure, a band is observed at 3300 cm^−1^ for the three COPs corresponding to the N-H bond stretching, present in the original porphyrin macro ring. Each COP exhibits characteristic IR bands in the 1600–1000 cm^−1^ range, that differ from those present in the same range in the spectra of the co-monomers. One interesting finding is that the COP spectrum indicates that a proportion of the pinacolate or boric acid groups of the starting cross-linkers are still present in the final COP. To illustrate this point, Figure S2 shows the stretching of the C-H bond of pinacolate at 2980 cm^−1^ and the O-H stretching signal of the boric acid functional groups at 3450 cm^−1^. It is proposed that these residual functional groups are on the surface of the material, due to the consumption of the Porph-Br_4_ reagent, and they could even be used for further post-synthetic modification of the COP surface.

Thermogravimetric analysis performed under air was used to determine the thermal stability of the materials and the possibility of contamination by inorganic salts (see Figure S3). It was found that the three materials exhibit a sharp combustion decomposition step at a temperature of 500 °C under dynamic heating conditions. This decomposition temperature is, however, slightly higher as the linker rigidity increases. Considering a weight loss over 91% in the case of COP–1 and COP–2 and over 95% in case of COP–3, the minor residue in thermogravimetry can be attributed to graphitization of the polymer, and, to a lesser extent, the inorganic impurities introduced during the synthesis of the materials.

**Figure 2:**
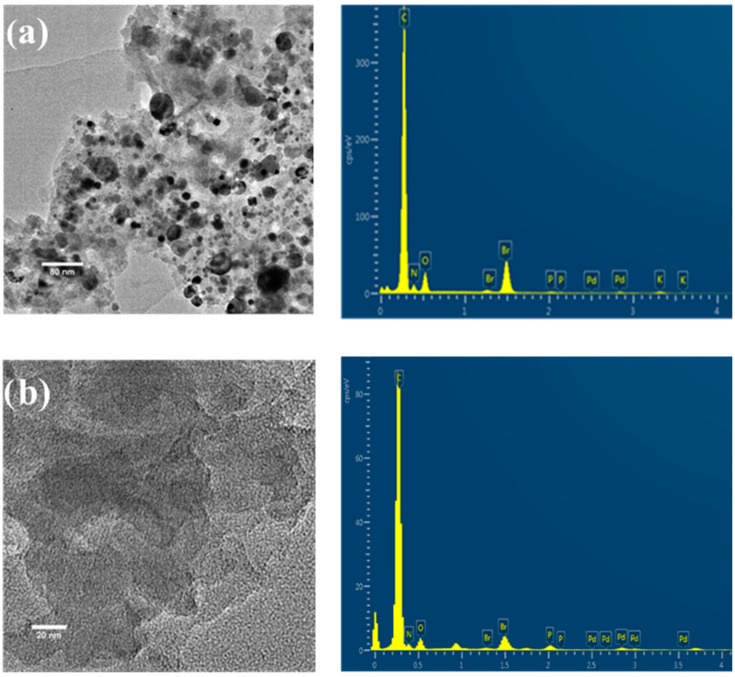
TEM images and EDX of: COP–3 before (**a**) and after (**b**) Pd NPs removal.

After the synthesis of COPs, it can be seen that the presence of Pd NPs is evident by the presence of higher contrast NPs in the TEM images (Figure 2a). Washing COPs with NaCN solution was shown to be an efficient process to remove Pd NPs as no more Pd NPs were observed in the TEM images (Figure 2b). However, although Pd NPs disappeared after the washing, atomic Pd was still detected in small quantities (0.8% *w*/*w*) with elemental analysis by EDX. This fact was corroborated with ICP elemental analysis after digestion with piranha solution. Since Pd NPs are not present in the COP samples, the detected Pd must be retained into the polymers in the form of metallic complex, trapped in the molecular structure during the polymerization process.

**Figure 3:**
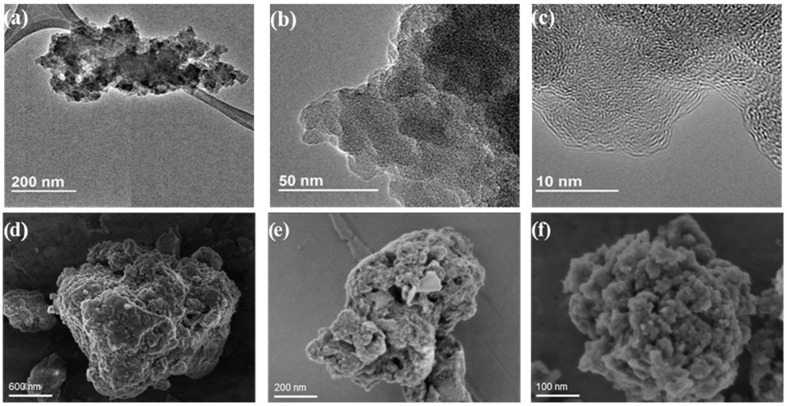
TEM images of COP–3 (**a**–**c**) and SEM images of COP–1 (**d**), COP–2 (**e**), and COP–3 (**f**).

The morphology of COP particles was analysed by electronic microscopy. Figure 3 shows TEM and SEM images of the three COPs. TEM images showing a feature of COP–3 with various magnifications indicate that some crystallinity is observed, suggesting stacking of the different monomers probably induced by weak π−π bonds between aromatic rings. According to SEM images, the three COPs were constituted by irregular particles with a wide distribution of particle size, going from small NPs of hundreds of nanometres to larger grains of a few micrometres. The planar molecular geometry of the cross-linker monomer in COP–1 is probably responsible in this sample for the presence of flat particles with observation of terraces. In contrast, COP–2 and COP–3 exhibit a particle surface with a granular shape, which could explain why the surface measured is higher in these samples. It is also noticeable that in all samples there was high porosity, from large pores of about 10 nm to others that were very small in the range of 1 nm.

**Figure 4:**
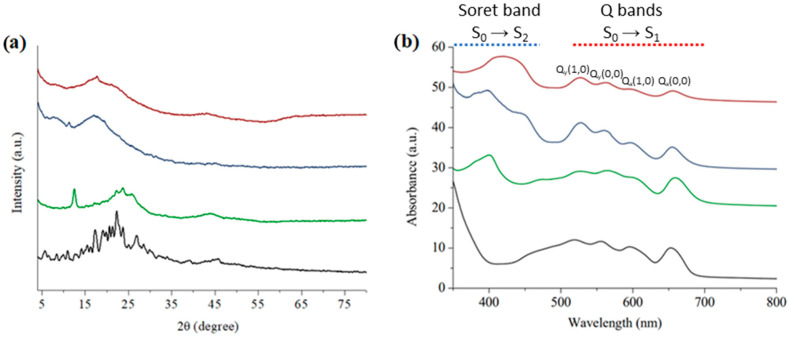
(**a**) X-Ray powder diffraction spectrum of: Porph-Br_4_ (black line), COP–1 (green line), COP–2 (blue line), COP–3 (purple line). (**b**) Solid state UV-Vis absorbance recorded for Porph-Br_4_ (black line), COP–1 (green line), COP–2 (blue line), and COP–3 (red line).

XRD patterns of the three COPs are shown in Figure 4a that indicate the materials have poor crystallinity and too broad bands; we can conclude that COPs made are mainly amorphous. The 3D COPs exhibit very broad XRD bands at angles about 7°, 17°, and 45° whereas COP–1 with a 2D structure has the same band at higher angles. COP–1 exhibit a XRD spectrum also with broad bands at diffraction angles of 15°, 20°, and 45°, however, from these bands other sharper peaks can be seen. In fact, these bands and sharp peaks are in the same position as recorded for initial free Porph-Br_4_ powder, in which, porphyrins are mainly packed together by π-π stacking. This fact could indicate for COP–1 that porphyrin moiety has some degree of stacking in certain regions of the molecular structure. This will be compatible with the planar geometry of the two co-monomers. In this sense, 3D inductors cross-linkers may induce lower π-π stacking of porphyrins while synthesis take place.

### 3.2. Photochemical Characterization

The presence of metal-free Porph-Br_4_ in COPs rules UV-Vis absorption spectroscopy in all samples showing very similar UV-Vis spectrum. Most important is that optical spectrum of these materials revealed they can harvest photons from 380 to about 680 nm, corresponding to a large percentage of the solar light spectrum. In the three COPs, absorption of the porphyrin component was, as expected, more intense and masked the absorption spectra of the other co-monomers that absorbed in the UV part of light spectra. The absorption spectra of all samples consist of a Soret band or B band, conventionally identified as S_0_→S_2_ transition [47,48], at around 340–415 with a shoulder at 440 nm in the case of COPs (see Figure 4b). Additionally visible in the UV-Vis spectra is the appearance of four bands between at 520 and 654 nm that are known as Q bands [47,48,49]. These bands are traditionally divided in two doublets Q_y_ (Q_y_(1,0) and Q_y_(0,0)) and Q_x_ (Q_x_(1,0) and Q_x_(0,0)) and are responsible for the S_0_→S_1_ transition [48]. The Soret band of solid Porph-Br_4_ was blue shifted and found at 340 nm in comparison to the COPs. This fact is explained because porphyrins molecules are highly compacted together due to π−π stacking among aromatic rings. This is something that could be easily confirmed by measuring the UV-Vis spectra in liquid phase of a very diluted acetonitrile solution of Porph-Br_4_ in which π−π stacking of the polyaromatic ring does not occur (see Figure S4a). Additionally, COPs Q bands have more intense absorption compared with the Soret band in original Poprh-Br_4_, that could be related to how the porphyrins are ordered in the polymeric molecular network. As is observed in Figure 4b, a noticeable bathochromic shift of 10 nm in the peak position corresponds to Q_x_(0, 0) transition on going from COP–3 to Porph-Br_4_, and is rationalized as increased resonative interaction of negative charge density around the N atom of pyridine with porphyrin macrocycle on going from Porph-Br_4_ to COP–3 [48].

Regarding the photocatalytic activity that is the subject of this work, one important piece of information is the band gap energy of the excited state of the COPs. The energy band alignment with respect to the redox potentials required for the photocatalytic water splitting (PWS) is a pre-requisite that must be met in order for the reaction to occur. Crucial for a PWS is not only achieving a certain redox potential of about 1.23 eV plus and over potential to overcome defects and other competitive reactions, but also the correct alignment of the frontier orbitals with respect to the energy of the two half reactions. The HOMO-LUMO energy band gap was estimated by optical spectroscopy evaluating the zero-zero vibrational state excitation energy E^0,0^ value from the interception of the excitation and the emission spectra. Specular emission and excitation spectrum are normalised and the meeting point between the two traces corresponds to the energy of the ground state and excitation state transition of the materials. Figure S5 in the supporting information shows excitation and emission spectrums, while Table 2 gathers these energy values. Analysis of these energy values led us to conclude that the three COPs are able to generate hydrogen and oxygen photocatalytically. The series of energy values for COP–1, COP–2, and COP–3 are also in the range of data reported in the literature for related porphyrins in solution [41,44]. According to this, the main photocatalytic properties of these COPs seem to derive from the porphyrin units, with some tuning due to the cross-linker unit used in the COP preparation.

### 3.3. Nanosecond Time-Resolved Transient Absorption Spectroscopy (TAS)

With the aim of understanding the photochemistry of COPs, transient excited state was characterized after excitation with a 415 nm nanosecond laser pulse; this wavelength will induce photoexcitation of porphyrin moieties. At the nanosecond scale, we can monitor deactivation of the excited triplet to the ground state and the generation and decay of the charge separation state. Initially, we wanted to figure out how polymerization affects the dominant Porph-Br_4_ photophysics in COPs. To do so, we recorded the TAS spectra of Porph-Br_4_ monomer and COP–3. The TAS spectrum of Porph-Br_4_ has similarities to that of Porph-Br_4_ ground state absorbance, in which an intense peak appears at 440 nm and other less intense peaks between 520 nm and 700 nm (see Figure S6). Yeduru et al. [48], who have done femtosecond TAS of very similar porphyrins, conclude that TAS spectrum is the result of three transitions that occur in less than a few nanoseconds. First, the excitation of electrons from S_0_ to S_2_; then, Q bands appear due to internal relaxation of electrons from S_2_ to S_1_ (Q bands); and finally, the intersystem crossing from Q_x_ to triplet excited state occurs. In the TAS spectrum of COP–3, the most intense band has blue shifted with a maximum at 390 nm meaning that the energy gap of S_2_ to S_0_ transition has increased compared with the pristine Porph-Br_4_ moiety. This fact agrees well with our calculations of E_op_^0,0^ and E_elec_^0,0^ that shows higher values for COPs with respect to Porph-Br_4_. Furthermore, the lifetime of excited state in COP–3 was about 65 ns, much shorter than that of the parent Porph-Br_4_ triplet excited state that was 1.7 μs. In fact, it has the same lifetime as when Porph-Br_4_ triplet excited state is quenched by O_2_ (see Figure S7). This shorter lifetime indicates that the triplet excited state in COP–3 is quenched due to fast energy transfer between porphyrin units in the COP framework as a consequence of porphyrin π–π interaction. Based on these results, it is proposed that one strategy to increase photocatalytic activity would be to increase the lifetime of the excited state by increasing in the polymer molecular structure steric hindrance to porphyrin interaction. Decay kinetics in COPs show some differences in the region between 350–450 nm (S_0_→S_2_) in comparison to Porph-Br_4_. In this region, COPs exhibited an excited state decay with two kinetics: a short one that corresponds to triplet excited state (65 ns) and another kinetic with a longer lifetime (0.5–1.5 μs), corresponding to delocalization of photogenerated charges over polymer framework (see Figure S8). This fact explains why COP–1 with an aromatic, planar, and conjugated molecular structure that facilitates charge delocalization from porphyrins to pyrene moieties has a longer excited state lifetime (see Table S2). Finally, through quenching experiments with water, see Figure S9, we estimated a quenching constant of K_q_ = 5 × 10^6^ M^−1^; this value demonstrates that there exists a strong interaction between water and the excited state of COP–3. Thus, the excited state of our COPs could exchange charges with water molecules.

### 3.4. Electrochemical Characterization

Cyclovoltammetry (CV) measurements in acetonitrile allowed the determination of the reduction potential by scanning electrode voltage towards cathodic potential values for all samples. The results are summarized in Table 3 and the corresponding electrochemical plots are included in the supporting information (Figure S10). As can be seen in Table 3, the Porph-Br_4_ and the three COPs presented a reduction peak between −0.90 to −1.1 V. It is proposed that this reduction peak corresponds to the LUMO potential. Furthermore, a weak oxidation pair is observed at positive potentials between 0.8 and 1.3 V that corresponds to the HOMO electrochemical potentials. As can be seen, all polymers have slightly more reductive and oxidative redox potentials than Porph-Br_4_. Again, these small differences in potentials must be related to how porphyrins are packed when they are polymerized. It can be seen into photochemical properties of COPs, the redox behavior of all COP polymers is dominated by the porphyrin moiety. Although the behavior between them is relatively the same in dark conditions, small differences appear when electrodes were illuminated. In this sense, Porph-Br_4_ and 2D COP–1 show two reduction bands that might correspond to the loss of the hydrogens bonded to the pyrrole nitrogen of porphyrins. However, COP–2 and COP–3 samples show, in dark and under illumination, only broad reduction peaks that should include both reduction potential. Furthermore, it must be commented that under illumination, all CV plots experience a change in the intensity of reduction and oxidation peaks. Specifically, reduction peaks became more intense while oxidation peaks became attenuated in Porph-Br4 and COPs. Finally, we calculated the electronic band gap under our working conditions applying the following equation E_elec_^0,0^ = E_ox_ (V) − E_red_ (V) [50,51]. E_ox_ and E_red_ values were taken at the beginning of the reduction or oxidation peaks. It was found that the overall values obtained differ very little from the calculated through a Tauc plot from acetonitrile suspension. All calculated electronic band gaps give higher values compared with optical band gaps except initial Porph-Br_4_ that is smaller. We must take into consideration two things in band gap calculations: first, that electrochemical working conditions differ from the UV-Vis absorbance spectroscopy and second, Tauc plot calculations are more precise for metal oxides semiconductors. All these together should justify these small differences in values.

Photocurrent density experiments were carried out for all three COPs. The aim of doing these experiments is to confirm by electrochemical means that the materials are able to photogenerate charges when stimulated with solar-simulated light. In this sense, photoelectrodes of the COPs were made onto FTO conductive glass by “Doctor Blade” technique and tested in acetonitrile solution with 0.2 M TBAPF_6_ as an electrolyte applying a potential of 1.2V. The results in Figure S11 show that the three COPs are able to generate significant current after illumination, with COP–3 being the material that photogenerated the highest current density of all samples. Thus, the ability to photogenerates charges can be improved in COP with flexible and 3D molecular structure in COPs.

### 3.5. Characterization of Photogenerated Electron and Holes

After absorbing photons, photocatalysts can produce charge separation into electrons (e^−^) and holes (h^+^) that are then utilized to perform chemical reactions. Methanol is commonly employed in photochemical experiments as a hole quencher because is a good electron-donor agent. In this sense, methanol will improve the overall photocatalyst photocurrent by injecting electrons to holes (*COP–3(h^+^)*) (see reaction 1). The photocurrent was recorded after adding 50 μL of methanol, resulting in a considerable 4.5-fold photocurrent density enhancement in comparison to not adding methanol (see Figure S11). This increase is higher than that of the starting porphyrin photoelectrode, confirming that COP–3 has more capacity to exchange charges than COP–1, COP–2, and Porph-Br_4_. All these facts not only confirm that the materials are able to generate electrons and holes and thus produce current into the electrochemical set-up, if not also that charges generated upon illumination are able to migrate out of the polymers.

$$COP\text{-}3+h\nu \to COP\text{-}3^{*}$$

$$COP\text{-}3^{*}\to \;COP\text{-}3\left(h^{+}\right)+COP\text{-}3\;\left(e^{-}\right)$$

$$COP\text{-}3\;\left(h^{+}\right)+MeOH\left(l\right)\to COP\text{-}3+MeOH^{+}\;\left(g\right)$$

$$COP\text{-}3\;\left(e^{-}\right)\;\to electrode\left(\;e^{-}\right)+COP\text{-}3$$

**Reaction 1.** Hydrogen production reaction with the presence of MeOH of hole quencher.

Photocatalytic deposition experiments serve as evidence of the presence of photogenerated electrons, but can also be useful in locating the photoactive site where charge separation occurs in the photocatalyst. In this type of experiment, the metal compound ends photodeposited in the position where charges are located on the catalyst. For this purpose, we selected RuCl_3_ salts to perform this experiment. Initially, the Ru^3+^ cation gets reduced to Ru^2+^ and to insoluble RuO_2_ that precipitates on the photocatalyst employed. In the present case, irradiations were performed with simulated sunlight in the presence of aerobic oxygen as the electron acceptor. The process of photocatalytic deposition of Ru^3+^ to RuO_2_ deposition is shown in reaction 2. The photodeposited RuO_2_ was detected by high-resolution transmission electron microscopy that allowed us to measure the corresponding interplanar distance (see Figure S12).

$$COP\text{-}3+h\nu \to COP\text{-}3^{*}$$

$$COP\text{-}3^{*}\to COP\text{-}3\;\left(h^{+}\right)+COP\text{-}3\;\left(e^{-}\right)$$

$$COP\text{-}3\;\left(e^{-}\right)+Ru^{3+}\left(aq\right)\to \;COP\text{-}3+Ru^{2+}\left(aq\right)$$

$$Ru^{2+}\left(aq\right)+O_{2}\to \;RuO_{2}\left(s\right)$$

**Reaction 2.** Photocatalytic RuO_2_ photocatalytic deposition reaction.

### 3.6. Photocatalytic Water Splitting Experiments

**Figure 5:**
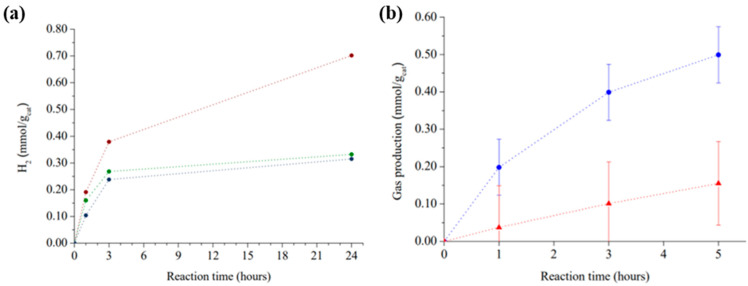
(**a**) PWS at 30 °C under simulated sunlight in presence of: COP–1 (●), COP–2 (●) or COP–3 (●). (**b**) PWS of COP–3, generation of hydrogen (●) and oxygen (▲).

Comparison of the temporal profiles of the photocatalytic hydrogen evolution without a sacrificial agent shows that there is significant hydrogen generation upon simulated sunlight in the presence of COP (see Figure 5a). The order of the photocatalytic activity for the three materials at the same working conditions was COP–3 > COP–2 > COP–1. These results reveal that a 3D molecular structure has benefits in the photocatalytic performance. For instance, COP–1 in 3 h generated 0.21 mmol·g^−1^ whereas COP–2 reached 0.27 mmol·g^−1^. Furthermore, it is interesting to notice that there exists a photocatalytic improvement when COPs had less rigid molecular structure, achieving the highest hydrogen production of 0.4 mmol·g^−1^. It is important to consider that porphyrin is the common moiety of the COPs and that the main difference between the materials is that COP–3 has the highest surface area and smaller pore size. Further, we must point out the high hydrogen production reached for COP–3; that is a remarkable value considering the absence of any metal co-catalyst and using simulated solar light. The second remarkable feature of the hydrogen production of COPs is that hydrogen evolution apparently gets reduced beyond 3 h. During photocatalytic experiments, it was found that if the reactor was not periodically sonicated, productivity H_2_ and O_2_ of the materials decreased. For this reason, we speculated that this could be due to hydrogen or oxygen filling the pores of the material, resulting in a quenching of the reaction. For COP–3, oxygen production is also observed, and it is plotted with the hydrogen production for the same reaction times in Figure 5b. After 3 h of photocatalysis, the molar ratio of hydrogen and oxygen is below the expected stoichiometric molar ratio for overall water splitting as it is H_2_:O_2_ 2.9:1. However, the existing difference due to a lower production or detection of O_2_ could be ascribed to one of the following factors: low sensibility of the MicroGC equipment to O_2_ because of the low thermal conductivity of the gas and the attempt of simultaneous detection of both gaseous products, or oxygen being consumed partially inside the pores due to gas adsorption properties of the COPs. Preliminary controls by irradiation of the dry powders in argon for prolonged periods (5 days) COPs did not allow the detection of hydrogen or any gas evolving from the solids. Characterization of these powders by XRD and UV-Vis absorption spectroscopy did not reveal any change in the intensity or position of the absorption bands, supporting that all COPs are stable under irradiation. In this way, it is unlikely that the hydrogen detected comes from COP decomposition in a few hours, since the materials were stable for much longer periods of light exposure without evolving H_2_ in the absence of H_2_O.

It is interesting to make a small comparison of our results with recent publications of photocatalytic systems similar to ours, formed by some kind of organic framework such as MOF, COF, and g-C_3_N_4_. In the case of MOF, a recurrent issue of this material is the low resistance of the metallic node to suffer water corrosion and metal leaching. However, recently, publications have found more robust MOFs in water solutions such as Cabrero-Antonino et al. [52], who showed that in MIL-125(Ti)NH2 plasma treated MOF, PWS efficiency was enhanced if the defect is generated into the MOF framework, producing 83 μmol of H_2_·g^−1^ after 10 h (8.3 μmol·g^−1^·h^−1^). IEF-11 was another interesting MOF base on Ti(IV) metal nodes showing outstanding photocatalytic performance reaching 672 μmol of H_2_·g^−1^ after 22 h (31 mmol·g^−1^·h^−1^) [53]. In the field of COFs, there are several publications exploring the synthesis of porphyrin-based materials, but only a few are also related to PWS. For instance, Rufan et al. [54] studied the structure-property-activity relationship in isostructural porphyrin COFs. In this context, imine-bonded H_2_Por-DETH-COF reached 80 μmol·g^−1^·h^−1^ upon visible light irradiation. Nevertheless, they employed co-catalysts as Pt and Ru, sacrificial agents, and they performed the reaction in a phosphate buffer; all three factors contribute to boost photocatalytic hydrogen evolution, whereas we performed the photocatalysis in pure water and in a metal-free environment. Other authors [55] prepared nanodisks derived of exfoliated imine bonded porphyrin COF that were able to produce 5 μmol·g^−1^·h^−1^ under visible light. Other materials, such as g-C_3_N_4_, have gained interest in the last 10 years and have been used for many photocatalytic applications due to their robustness. For example, Yuan et al. prepared g-C3N4 following different strategies to enhance hydrogen evolution reaction with the presence of sacrificial agent, reaching a maximum production rate of 20 μmol·g^−1^·h^−1^. Other examples include the work published by Chen et al. [56], who showed that under visible light and without a sacrificial agent, bulk g-C_3_N_4_ was able to produce 10 μmol·g^−1^·h^−1^ whereas, nanostructured g-C_3_N_4_ was able to reach ten times more. Although this is a remarkable result, considering the use of visible light, this catalytic efficiency was reached with the help of co-catalysts such as Pt and Ir. In this sense, the photocatalytic H_2_ evolution rate of COP–3 of 133 μmol·g^−1^·h^−1^ is competitive considering that the photocatalytic experiments were performed in the absence of co-catalysts and in ultrapure water without a sacrificial agent. In summary, our COPs present two advantages with respect to the discussed materials: molecular structure is more robust against hydrolysis and temperature due to carbon bonding of monomers in comparison to MOFs and COFs, and porphyrin-based COPs show enhanced visible absorption with respect to traditional MOFs and g-C_3_N_4_.

**Figure 6:**
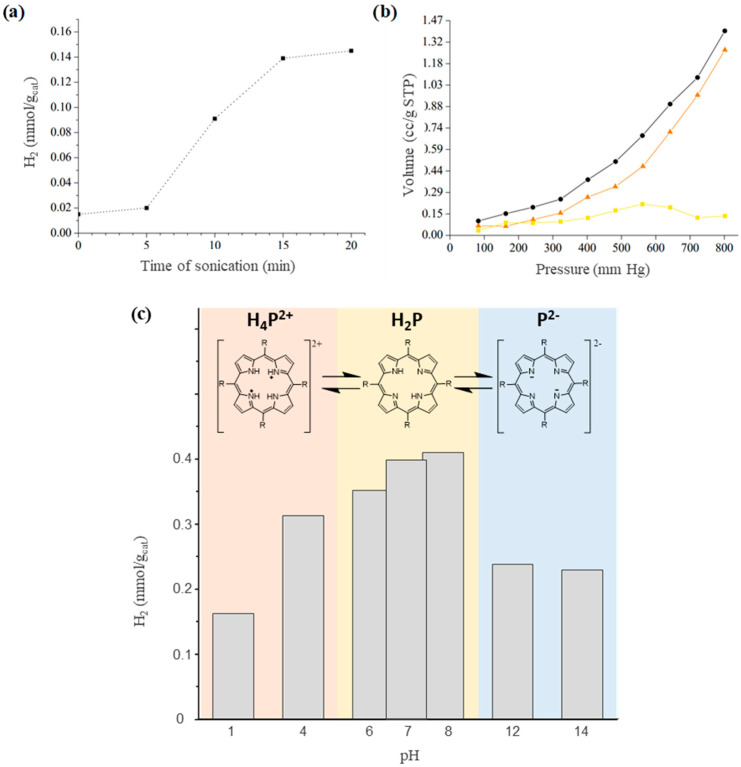
(**a**) Optimization of sonication time experiment. (**b**) Hydrogen chemisorption (■), physisorption (▲) and combined absorption (●) of COP–3. (**c**) Hydrogen production per gram of COP–3 after PWS reaction at different pH.

As commented earlier, before gas sampling it is necessary to sonicate the reactor to remove the adsorbed H_2_ into the COPs because we detected that the amount of H_2_ in the reactor gas phase increases significantly after sonication. For this reason, we were interested to know the optimum sonication time to measure most of the H_2_ produced. To do so, first we did 1 h of PWS employing COP–3 as photocatalyst, and afterwards the photoreactor was submitted to sonication for 20 min while gas samples were measured every 5 min. Figure 6a presents the influence of sonication time on the amount of measured hydrogen in the head space. It is proposed that the hydrogen evolved in the photocatalytic reaction is adsorbed into the internal pores of the COPs rather than evolving into the head space volume of the photoreactor. Thus, the higher amount of hydrogen was measured after 15–20 min sonication. To obtain some evidence in support of photogenerated hydrogen remaining adsorbed into the pores and not being released to the head space until sonication is performed, a series of hydrogen adsorption measurements at 40 °C under relevant pressure conditions were carried out for the COP–3 sample, the best performing photocatalyst. A difference between physiosorbed and chemisorbed hydrogen was experimentally determined by heat of absorption (see Figure 6b). As can be seen there, COP–3 absorbs a measurable hydrogen volume even at 40 °C and under pressures in the range of 80 to 800 mm Hg. Most of the volume corresponds to physiosorbed hydrogen, particularly at higher pressures. It must also be taken into consideration that after cleaning the material with cyanide and removing the Pd NPs, there is still <1% *w*/*w* of atomic palladium, which is probably responsible for the small amount of chemisorbed hydrogen. Since the volume of absorbed hydrogen per gram of catalyst is over 1.3 cc/g, this adsorption explains why, during the photocatalytic generation experiments, a small amount of the total hydrogen was detected until hydrogen desorption was promoted by ultrasounds. In this context, if we reduce physisorption capacity of COPs, we would minimize recombination of H_2_ and O_2_ gases within the pores and thus enhance photocatalytic activity.

Further study of the photocatalytic mechanism led to the analysis of the dependence of the PWS performance on pH for COP–3 (see Figure 6c). Neutral porphyrin ring (H_2_P) can protonate or deprotonate the nitrogen present in the pyrrole rings that form the porphyrin moieties and thus, this causes a change in their photophysical and electrochemical properties. It is described in the literature that at pH under 4.5, meso-porphyrins protonate (H_4_P^2+^), and at pH above 10, porphyrin rings lose the two initial protons (P^2−^) [57,58]. After screening pH dependence in the hydrogen formation rate, it has been obtained that the optimal pH for water splitting reaction is between pH 6 and 8. At this range of pH, the main species present in the porphyrin moiety of COP–3 is H_2_P. Moving to acidic pH, H_2_P passes to H_4_P^2+^ and the photocatalytic performance drops dramatically. This agrees with the computational studies that other authors have done recently, where they claim that crowding inner hydrogen atoms attached to the pyrrole nitrogen atoms leads to distortion of the porphyrin core affecting the molecular flexibility [59]. Moreover, other authors have revealed that protonation at the pyrrole nitrogen atoms caused positive shifts of the reduction potentials of derived porphyrins meaning that porphyrin moieties have less reduction strength [57,60]. Additionally, it has been reported that protonation of H_2_P to H_4_P^2+^ leads to a decrease in the overall quantum efficiency and lifetime of the excited state, which also explains its poor photocatalytic performance [59]. In the case of high basic pH, H_2_P pass to P^2−^, and the decrease of photocatalytic efficiency is less relevant but considerable. This small loss in efficiency can be explained if we consider that at this pH, we are favoring oxidation of water that is the bottle neck of PWS reaction, whereas acidic pH promotes water reduction. Moreover, considering that any of the cross-linkers suffer any changes due to working pH conditions and COPs do so, we can conclude that the photocatalytic active site into our COPs is located in the porphyrin ring. In summary, as most efficient photocatalysis occur at slightly basic pH, PWS in porphyrin-based COPs must involve both neutral porphyrin and the basic form of the porphyrin ring, allowing the porphyrin ring to interconvert in both forms during photocatalysis (see Scheme S2).

**Figure 7:**
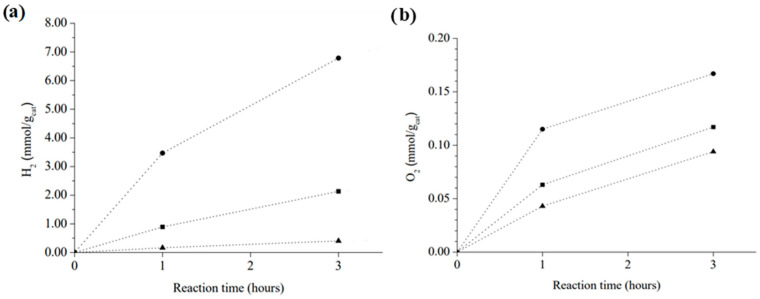
Photocatalytic hydrogen generation of COP–3 in the presence of sacrificial agents. (**a**) Amounts of methanol added: nothing (▲), 2.4 mmol (■) or 24 mmol (●). (**b**) Amounts of (NH_4_)Ce(NO_3_)_6_ added: nothing (▲), 1 mmol (■) or 3 mmol (●).

To understand the reactivity of excited states of these materials, it is crucial to study photocatalysis under the presence of quenchers of holes or electrons. In this way, we tested COP–3 hydrogen generation performance into a 20% *v*/*v* methanol solution. Methanol is often utilized as an electron donor because it reacts easily with photogenerated holes leaving excited electrons on the photocatalyst able to reduce water to hydrogen. Eventually, methanol oxidizes, forming radical cation of methanol (see reaction 2). Figure 7a shows that, as methanol concentration rises, hydrogen production increased. In fact, after adding 24 mmol of methanol, 17-times higher H_2_ production (3.48 mmol of H_2_·g^−1^ in 1 h) was reached after 3 h of reaction compared with the same reaction conditions without methanol. This observation indicates that hole consumption is limiting hydrogen evolution, that is substantially higher when electron donor compounds better than H_2_O are added to the system.

To shed light on the understanding of the reactivity of photogenerated electrons, additional experiments were performed, adding increasing amounts of ammonium cerium (IV) nitrate to the 20 mL of water in the photoreactor. The active quencher will be the Cerium cation, specifically Ce^IV^ that will act as the electron acceptor passing to Ce^III^ (Figure 7b). The addition of Ce^IV^ resulted in a considerable decrease in the production of hydrogen that, at the highest Ce^IV^ concentration (3 mmol of Ce^IV^ in 20 mL water), diminished by a factor of almost ten and evolution of oxygen increasing to 0.167 mmol of O_2_/g_cat_ in 3 h. This oxygen amount was about two times higher than that measured in overall water splitting. Under these favourable conditions, oxygen production grows with the amount of Ce^IV^ added to the solution, while hydrogen generation is decreased progressively. The observation of some residual hydrogen evolution indicates that Ce^IV^ was unable to completely quench all the photogenerated electrons, probably due to diffusion limitations of this quencher through the polymer pores. However, the notable diminution of hydrogen evolution together with the increase in oxygen concentration confirm the expected ability of Ce^4+^ to capture electrons. Selection of the Ce^4+^ salt seems to be relevant in this experiment, since (NH_4_)_2_Ce(NO_3_)_6_ does not change the solution pH significantly, whereas Ce(NO_3_)_4_ notably decreases the solution pH and oxygen formation is lower than with the ammonium cerium salt. Moreover, during the experiment CeO_2_ was deposited over COP–3 suggesting that Ce^4+^ to CeO_2_ reaction is occurring on the surface.

All the photocatalytic experiments with quencher together indicate that water oxidation is notably more difficult, being the limiting half reaction. In this sense, it is necessary to improve the oxidation capacity of porphyrin-based COPs. A way to do it would be by enhancing accessibility of charges promoting charge mobility across the polymeric molecular network. Another method to improve the photocatalytic activity would be to avoid the chemi- and physisorption of H_2_ or O_2_ on the surface of the COPs, so less photogenerated charges would be lost by recombination with these gaseous molecules.

**Figure 8:**
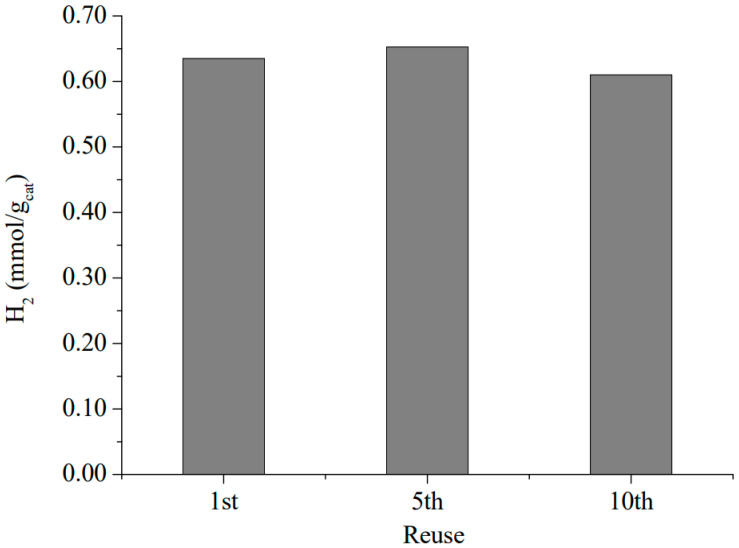
Reusability test of COP–3. For the sake of clarity, the figure shows the hydrogen generation in the first, fifth, and tenth reuse experiment of 6 h.

The stability of the COP–3 as a photocatalyst for hydrogen evolution from pure water was ascertained by performing ten consecutive reuses of the same material; see Figure 8 that summarizes the reusability test. Almost identical temporal profiles for hydrogen evolution are measured for these experiments after 10 uses of 6 h. The 60 h used sample did not reveal any fatigue regarding photocatalytic activity and no changes in XRD peaks and UV-Vis absorption bands were perceived, thus supporting the stability of the photocatalyst under irradiation conditions.

Importantly, considering porphyrins as the photocatalytic sites, the total accumulated turnover number for hydrogen production was 5.98. The turnover was calculated by estimating the catalytic active sites as the total amount of porphyrin in mols that has each COP per gram, according to elemental analysis. Perhaps this estimation of active sites is too generous since not all porphyrins will be available; in this context, the turnover number could be even higher. This turnover number together with COP stability support that the process is catalytic and not based on stoichiometric consumption of porphyrins during the hydrogen evolution.

## 4. Conclusions

In this work, we have achieved the synthesis of photoactive and stable metal-free COPs based on porphyrins via Suzuki–Miyaura coupling. The carbon-carbon bonds between the monomers favor the stability of the COPs in very acidic or basic solutions, high temperatures (<450 °C), and organic solvents. The dimensionality and rigidity of the molecular structure of COPs can be tuned with the election of the cross-linkers resulting in significant impacts in surface area and pore size, transient excited state kinetics, photoluminescence quantum yield, photocurrent density, and redox potentials, thus affecting overall photocatalytic performance. Porphyrin-COPs showed optical band gap and electronic band gap in the range of 2 and 2.3 eV, respectively, which is sufficient to perform OER and HER reactions. In comparison with initial porphyrin moiety, COPs time-resolved transient absorption spectroscopy data demonstrate that there is a decrease of decay lifetime and changes in kinetics. Particularly, in the region corresponding to the Soret band transition (350–450 nm), they have a decay with two kinetics and a strong interaction with H_2_O molecules. Additional photochemical and electrochemical experiments have revealed that COPs can generate charge separation upon light irradiation and then inject these charges to molecules in the medium. The best photocatalytic performance was carried out by COP–3, made of flexible tetrahedral cross-linkers, reaching a hydrogen production of 0.4 mmol H_2_ × g_COP–3_^−1^ in overall water splitting after 3 h of irradiation with simulated sunlight. Further, COPs showed their best photocatalytic activity in pH ranges between 6 and 8, thus we have proposed that the photocatalytic mechanism must involve neutral and basic forms of porphyry rings. Photocatalytic quenching experiments proved that porphyrin-based COPs still have a lot of potential to develop in order to enhance HER and OER, and one way to do it would be to avoid chemi- and physisorption of the produced gases. After ten reuses, we achieved a turnover of approximately six, demonstrating that overall water splitting is occurring by photocatalytic means without showing any fatigue in its photocatalytic efficiency.

## Figures and Tables

**Table 1:** Nitrogen, carbon, and hydrogen percentages obtained from the combustion chemical analysis of COP–1, COP–2, and COP–3. Surface area and pore size for the three COPs estimated by isothermal N_2_ adsorption are provided.

Sample	%N	%C	%H	BET Surface Area (m^2^/g)	Pore Size (nm)
COP–1	5.418	80.250	4.089	43	13.9
COP–2	4.962	76.820	3.967	146	6.2
COP–3	5.059	75.902	4.307	638	4.4

**Table 2:** Photochemical properties in acetonitrile of the Porph-Br_4_ monomer and the three COPs under study.

	Absorption (nm) *		Emission (nm) **			
Sample	S Band	Q_x_ (0,0)	Q_x_ (0,0)	Q_x_ (0,1)	E_op_^0,0^(eV) ***	r**Φ**_PL_ (%) ****
**Porph-Br_4_**	340	652	652	724	2.023	-
**COP–1**	415	651	649	720	2.049	7.1
**COP–2**	418	646	660	730	1.931	4.6
**COP–3**	422	641	654	723	2.013	48

* Absorbance UV-Vis spectrum measured in solid state ** Photoluminescence of acetonitrile suspension recorded exciting at 414 nm for all samples, see photoluminescence and excitation spectrums in Figure S5. *** Calculated utilizing the value related to the intersection crossing of excitation and emission spectrum of samples and equation $E=h\cdot \frac{c}{\lambda }$. **** Relative photoluminescence quantum yield (r**Φ**_PL_) calculated related to the initial Porph-Br_4_.

**Table 3:** Summary of the electrochemical properties of the Porph-Br_4_ monomer and the three COPs. Samples measured in acetonitrile utilizing TBAPF_6_ 0.2 M as electrolyte.

Sample	E_op_^0,0^ * (eV)	E_red_ (V)	E_ox_ (V)	E_elec_^0,0^ ** (eV)	Current Density (mA/cm^2^)
Porph-Br_4_	1.96	−0.95	0.81	1.76	0.186
COP–1	2.10	−1.03	1.28	2.31	0.028
COP–2	1.91	−0.92	1.24	2.12	0.038
COP–3	2.05	−0.95	1.29	2.24	0.247

* Calculated utilizing the value related to the intersection crossing of excitation and emission spectrum of samples and equation $E=h\cdot \frac{c}{\lambda }$. ** Calculated by equation E_elec_^0,0^ = E_ox_ (V) − E_red_ (V).

## Data Availability

Not applicable.

## Supplementary Materials

The following supporting information can be downloaded at: https://www.mdpi.com/article/10.3390/nano12183197/s1, additional experimental information, such as, synthesis of 2,2′,7,7′-tetrakis(4,4,5,5-tetramethyl-1,3,2-dioxaborolan-2-yl)-9,9′ -spirobi[fluorene], synthesis of COPs, UV-Visible spectroscopy, fluorescence emission and excitation spectroscopy, calculation of the relative photoluminescence quantum yield (r**Φ**_PL_), Experimental determination of the singlet energy gap, transient absorption spectroscopy (TAS) measurements, photocatalytic deposition, cyclic voltammetry and photocurrent measurements, photocatalytic water splitting; Figure S1: ^1^H NMR spectrum of 2,2′,7,7′-tetrakis(4,4,5,5-tetramethyl-1,3,2-dioxaborolan-2-yl)-9,9′-spirobi[fluorene]; Scheme S1: Preparation route of the three COPs; Table S1: Porphyrin and cross-linker molar percentages and ratios in COPs; Figure S2; FT-IR spectrums of monomers and COPs; Figure S3: Thermogravimetric analysis performed under air of COPs; Figure S4: Superposed absorption UV-Vis spectrums in acetonitrile; Figure S5: Fluorescence emission spectra (black line, λex = 415 nm) and excitation spectra (red line, λem = 653 nm) recorded in acetonitrile; Figure S6: Nanosecond time-resolved transient absorption spectra and decays of Porgf-Br_4_ and COPs; Figure S7: Decay of the transient excited state of Porph-Br_4_ before and after purging with O_2_; Figure S8: Comparison of transient excited state decay at 380 nm of COPs; Figure S9: Summarizes quenching data for COP–3 with aliquots of water; Figure S10: Cyclovoltammetry in acetonitrile solution from 1.8 V to −1.8 V, under dark or solar simulated light.; Figure S11: Light on-off photocurrent density response of Porf-Br_4_ and COPs in acetonitrile solution; Figure S12: High resolution transmission electron microscopy (HR-TEM) imaging of resulting COP–3 after photodeposition of Ru.; Scheme S2: Proposed photocatalytic mechanism for porphyrin-based COPs. Table S2. summarizes the decay kinetic of three COPs.
