# Supplementary material for: Photocatalytic Water Splitting Promoted by 2D and 3D Porphyrin Covalent Organic Polymers Synthesized by Suzuki-Miyaura Carbon-Carbon Coupling
Source: Nanomaterials (Basel). 2022 Sep 14;12(18):3197. doi: 10.3390/nano12183197 (PMC9503735; doi:10.3390/nano12183197)
Supplement: Supplementary file 1 [file nanomaterials-12-03197-s001.zip › nanomaterials-1876499-supplementary.pdf]

SUPPORTING INFORMATION

PHOTOCATALYTIC WATER SPLITTING PROMOTED BY 2D AND 3D PORPHYRIN COVALENT ORGANIC POLYMERS SYNTHESIZED BY SUZUKI-MIYAURA CARBON-CARBON COUPLING

María Novoa-Cid[1,2], Arianna Melillo[1], Mercedes Alvaro[1], Belén Ferrer[1] and Herme G. Baldovi[1,2†]

[1] Department of Chemistry, Universitat Politècnica de València, 46022 Valencia, Spain;

[2] Instituto de Tecnología Química CSIC-UPV, Universitat Politènica de Valencia, 46022 Valencia, Spain

[†] Correspondence: hergarba@cam.upv.es

TABLE OF CONTENTS

Additional experimental information: synthesis of 2,2',7,7'-tetrakis(4,4,5,5-tetramethyl-1,3,2-dioxaborolan-2-yl)-9,9'-spirobi[fluorene], synthesis of COPs, UV-Visible spectroscopy, fluorescence emission and excitation spectroscopy, calculation of the relative photoluminescence quantum yield ($r\Phi_{PL}$), experimental determination of the singlet energy gap, transient absorption spectroscopy (TAS) measurements, photocatalytic deposition, cyclic voltammetry, photocurrent density measurements and photocatalytic water splitting**.**

Figure S1. ^{1}H NMR (400 MHz, $CDCl_3$, ppm) spectrum of 2,2',7,7'-tetrakis(4,4,5,5-tetramethyl-1,3,2-dioxaborolan-2-yl)-9,9'-spirobi[fluorene].

Scheme S1. Preparation route of the three COPs.

Table S1. Porphyrin and cross-linker molar percentages and ratios of COP-1, COP-2 and COP-3 calculated from combustion chemical analysis data.

Figure S2. FT-IR of monomers and COPs.

Figure S3. Thermogravimetric analysis performed under air of COPs.

Figure S4. Superposed absorption UV-Vis spectrums in acetonitrile of Porph-Br_4 and COPs.

Figure S5. Fluorescence emission spectra (black line, λex = 415 nm) and excitation spectra (red line, λem = 653 nm) recorded in acetonitrile.

Figure S6. Nanosecond time-resolved transient absorption spectrums and decays of 5,10,15,20-(tetra-4-bromophenyl)porphyrin and COP-3, in acetonitrile solution.

Figure S7. Decay of the transient excited state of Porph-Br_4 before and after purging with O_2.

Figure S8. Comparison of transient excited state decay at 380 nm of COPs. **Table S2** summarizes the decay kinetic of three COPs.

Figure S9. Summarizes quenching data for COP-3 with aliquots of water.

Figure S10. Cyclovoltammetry in acetonitrile solution from 1.8 V to -1.8 V, under dark or solar simulated light.

Figure S11. Light on-off photocurrent density response of Porf-Br4 and COPs in acetonitrile solution

Figure S12. High resolution transmission electron microscopy (HR-TEM) imaging of resulting COP-3 after photodeposition of Ru.

Scheme S*2*. Proposed photocatalytic mechanism for porphyrin-based COPs.

Additional experimental information.

Synthesis of 2,2',7,7'-tetrakis(4,4,5,5-tetramethyl-1,3,2-dioxaborolan-2-yl)-9,9' -spirobi[fluorene]. A mixture of 2,2',7,7'-tetrabromo-9,9'-spirobi[fluoreen] (1.0 g, 1.6 mmol), bis(pinacolato)diboron (3.0 g, 11.8 mmol), [1,1′-bis(diphenylphosphino)ferrocene]-dichloropalladium(II) (60 mg, 0.082 mmol), and potassium acetate (1.5 g, 15.3 mmol) in 1,4-dioxane (20 mL) was stirred at 75 °C for 72 h under N_2. The reaction mixture was then cooled to room temperature and quenched by water addition. The reaction mixture was extracted with four aliquots of ethyl acetate (20 mL). Then the organic layer was washed with brine, dried over anhydrous Na_2SO_4 at 120 °C for 24 h, and concentrated in a rotary evaporator to about 2 mL. The residue was purified by a Celite column to remove the catalyst, vacuum solvent concentration and silica gel column chromatography (hexane:ethyl acetate 1:4). Finally, the product was dried with a rotary evaporator and to obtain a white solid. Then, the crystals were purified with 3 mL of hot CH_2Cl_2 and cooled down for 3 days at 4 ^oC. Later, crystallization time, large crystals were collected and washed by filtering with cold methanol. Finally, the white solid was crushed and dried at 100 ^oC for 12 h to obtain 470.8 mg, 36.3% of the tetrakis borolane. ^1H NMR spectrum of 2,2',7,7'-tetrakis(4,4,5,5-tetramethyl-1,3,2-dioxaborolan-2-yl)-9,9'-spirobi[fluorene] is shown in Figure S1.

Synthesis of COPs. The COPs employed in this work were prepared by microwave-assisted solvothermal method using Milestone´s ETHOS 1 Advanced Microwave Digestion Labstation. To synthesize COP-1, 150 mg (0.32 mmol) of pyrene-2,7-diboronic acid pinacol ester 96% were reacted with 150.5 mg (0.16 mmol) of Porph-Br_4 (pyrene:porphyrin molar ratio = 2:1), produced an intense violet material. COP-2 was prepared by reacting 150 mg (0.18 mmol) 2,2',7,7'-tetrakis(4,4,5,5-tetramethyl-1,3,2-dioxaborolan-2-yl)-9,9'-spirobi[fluorene] and 173.7 mg (0.18 mmol) of Porph-Br_4 98% (spirobi[fluorene]:porphyrin molar ratio = 1:1), resulting in a brownish powder. COP-3, was prepared reacting 150 mg (0.29 mmol) of tetra(4-hydroxyborylphenyl)methane (TFM) 95% and 272.9 mg (0.29 mmol) of Porph-Br_4 (TFM:Porphyrin molar ratio = 1:1), obtaining a brown-reddish powder. For all these syntheses, the monomers were mixed with 50 mg (0.071 mmol) of $PdCl_2(PPh_3)_2$ and introduced into a Teflon autoclave with 20 mL of distilled tetrahydrofuran and 1.5 mL of 1 M aqueous K_2CO_3 solution. The reactor was then purged with argon (Ar) for 20 min and placed into the Microwave Digestion Labstation. For the coupling reaction the microwave was programmed with the following heating rate of 85 °C for 5 h, 30 min and 95 °C for 1 h 30 min. The temperature inside the autoclave was controlled with a thermocouple. COPs purification was carried out in the following way: first, the solution was filtered and the insoluble solid washed with THF; the resulting COP was then added to 20 mL of a 1 M NaCN solution that was stirred at 60 °C for 12 h to remove Pd nanoparticles;

after that, the polymer was filtered again and washed with abundant water before overnight drying. Finally, the solid was washed for 24 h with a Soxhlet with CH_2Cl_2 to remove the organic impurities. After drying the solid overnight, the powders were activated at 150 °C under vacuum overnight before their use.

UV-Visible spectroscopy. Optical ultraviolet-visible absorption spectra were recorded with a Jasco V-650 spectrophotometer for dispersions of each COP or for a solution of Porph-Br_4 in acetonitrile. These spectras were recorded in quartz cuvettes of 10 × 10 mm with 4 mL capacity.

Fluorescence emission and excitation spectroscopy. Steady-state fluorescence or photoluminescence and excitation measurements were performed with a FLS1000 photoluminescence spectrometer (Edinburgh Instruments, Livingston, UK), equipped with an N-DMM double-emission monochromator, an N-G11 PMT-980 detector and a pulsed Xe lamp (450 W) as the excitation source. Experiments were carried out on a dispersion of each COP sonicated previously for 30 min or with a solution of Porph-Br_4 in acetonitrile. For comparison and relative quantum efficiency analysis, all samples were measured in quartz cells of 10 × 10 mm with 4 mL capacity adjusting the optical density to an absorbance of 0.3 at 415 nm corresponding to the B or Soret band. Fluorescence excitation spectra were recorded fixing the detector monochromator to the first emission peak wavelength while the excitation wavelength is scanned.

Calculation of the relative photoluminescence quantum yield (rΦ_{PL}). An acetonitrile solution of Porph-Br_4 adjusted to an absorbance of 0.3 at 415 nm was used as reference. Then, suspensions of COP-1 to COP-3 were prepared with same optical absorbance at 415 nm and afterwards their photoluminescence (PL) spectrum were measured upon 415 nm excitation. The areas of the fluorescence spectra of Porph-Br_4 and PL spectra of COPs were calculated. The integrated areas of the COP PL spectra were divided by Porph-Br_4 fluorescence area and the ratio multiplied by 100 to obtain the Φ_{PL} relative to original Porph-Br_4.

Experimental determination of the singlet energy. The energy of the singlet state was estimated with $E=h \cdot c/\lambda$, considering the crossing wavelength (λ) of the normalized emission and the excitation spectra of each sample COP suspensions and free Porph-Br_4 solution in acetonitrile.

Transient absorption spectroscopy (TAS) measurements. Experiments were carried out with a Nanosecond Laser flash photolysis system using an OPO Ekspla (EKS-NT342C-10) laser coupled with an UV extension (EKS-NT342C-SH-SFG) as the excitation pulse and an Edinburgh Instruments detection System (LP980) coupled with an ICCD camera (Andor iStar CCD 320T). All samples were measured suspended in acetonitrile after adjusting the optical absorbance to 0.4. TAS of Porph-Br_4 was recorded in solution with the same optical absorbance and solvent. TAS

were acquired upon 415 nm laser pulse excitation purging the cuvette with Ar for at least 5 min. Chemicals in the form of gas or liquid were used to selectively quench transient electronic excited species generated in COPs after laser excitation. O_2 gas was purged into the cuvette as triplet excited state and electron quencher. N_2O gas was purged into the cuvette as solvated electron quencher. Methanol (200 mL) was used as hole or cation quencher.

Photocatalytic deposition. A water suspension of 50 mL of 10^{-3} M $RuCl_3$ and 50 mg COP-3 was irradiated at the open air under the same conditions as photocatalytic water splitting for 30 min. After this time, the COP solid was filtered and washed with abundant water. Deposition of RuO_2 was determined by transmission electron microscopy and confirmed by EDX analysis.

Cyclic voltammetry and photocurrent measurements. Films of the Porph-Br_4 or any of the three COFs were prepared on a transparent fluorine-doped tin oxide (FTO) working electrode by "doctor blade" technique. For this purpose, pastes of the corresponding photoactive materials were prepared stirring and curing at 90 °C for 15 h a mixture of 100 µL of α-terpineol, 10 mg of the corresponding material and 1 mL acetone, until a dense paste was obtained. Finally, the prepared films were dried at 120 °C for 5 h. Electrochemical measurements were carried out in a single compartment quartz electrolytic cell with three electrodes using a platinum wire as counter electrode and an Ag/AgCl in 3M KCl mixture as reference electrode. Cyclic voltammetry tests were performed using acetonitrile as solvent and 0.2 M tetrabutylammonium hexafluorophosphate ($TBAPF_6$) solution as electrolyte. Photocurrent measurements were performed upon illumination with monochromatic light from a Xe lamp in the presence of methanol as quencher and using an acetonitrile solution of 0.2 M $TBAPF_6$ as electrolyte.

Photocatalytic water splitting. All the photocatalytic experiments were performed at least in triplicate. The presented data points correspond to the average of three independent measurements and the error bar corresponds to the standard deviation. The reaction was performed in a pressurised and closed quartz reactor (55 mL) in batch operation. Before each experiment, to activate the photocatalyst the adsorbed organic solvent was evacuated by heating at 150 ºC under vacuum overnight. Then, after cooling at room temperature, 20 mg of the polymer was suspended in 20 mL of milliQ H_2O (1 mg of photocatalyst per mL of milliQ H_2O) and submitted to sonication for 20 min. Finally, the system was purged with Ar flow for at least 20 min before starting the photocatalytic reaction. All reactions were performed with freshly prepared material. After purging the system, the photoreactor was charged with Ar at an absolute pressure of 1.5 bar and the suspension was irradiated under stirring. As light source was utilized a Newport solar simulator, setting the incident solar simulated light power at 100 mW/cm^2. The evolved gases from the different experiments were analysed by sampling 2.5 mL from the head space with a Hamilton syringe at different reaction times into an Agilent 490 Micro GC system (Molsieve 5 Å

column and TC detector) using Ar as carrier gas. Also, the influence of pH was studied using NaOH and HCl as base and acid to set the initial pH of water solution at the required value.

Optimization of sonication time experiment was carried out after 1 h of PWS, then reactor was sonicated for 20 min, while gas content within the reactor was measured periodically every 5 min.

Sequentially photocatalytic experiments with sacrificial agents were carried out adding specific concentrations of methanol (0, 2.4 and 24 mmol) or $(NH_4)_2Ce(NO_4)_6$ (0, 1 mmol and 3 mmol) were independently added to the photocatalytic suspension as hole or electron trapping probes, respectively.

Reusability tests were carried out without removing the photocatalyst from the photoreactor but submitting the system after each reaction to 20 min Ar purging, 15 min sonication and repeating 20 min argon purging. Before starting a consecutive reuse, the absence of detectable hydrogen at the initial time was confirmed. Each reaction took 6 h, so the total irradiation time for the ten reuses was 60 h.

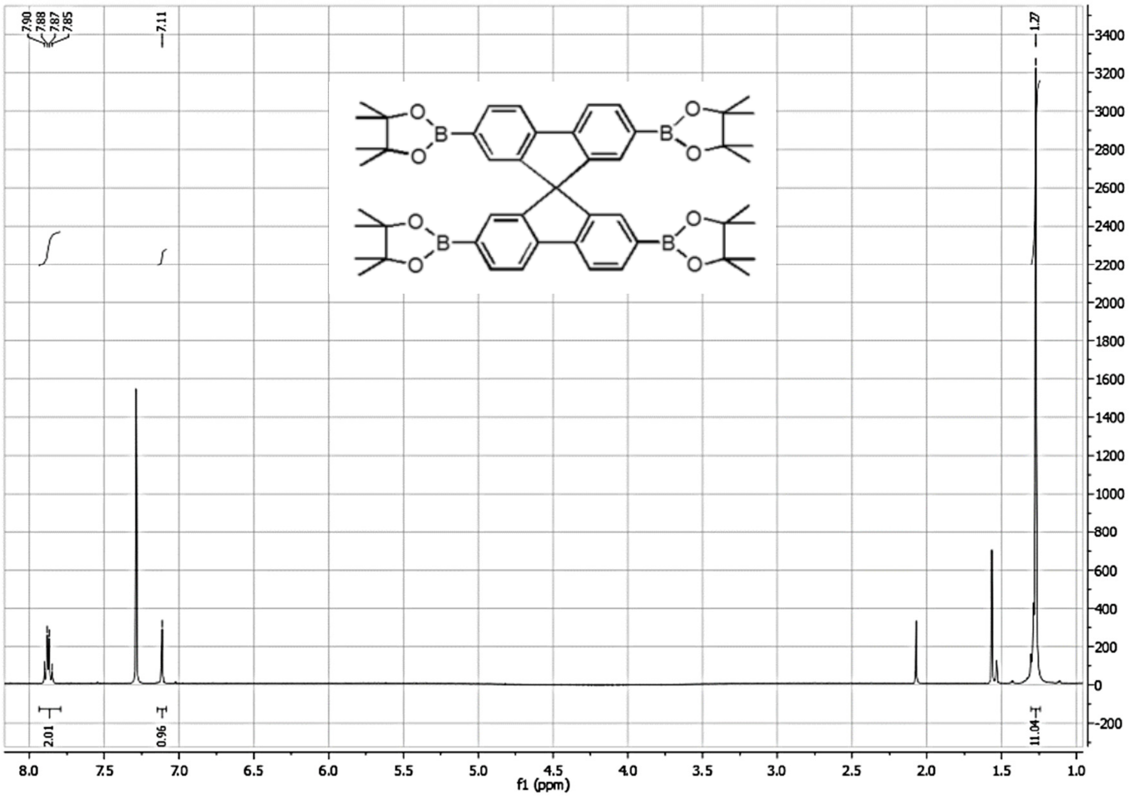

Figure S1. ^{1}H NMR (400 MHz, $CDCl_3$, ppm) spectrum of 2,2’,7,7’-tetrakis(4,4,5,5-tetramethyl-1,3,2-dioxaborolan-2-yl)-9,9’-spirobi[fluorene]: δ 7.85 (m, 8H), 7.09 (s, 4H), 1.25 (s, 48H).

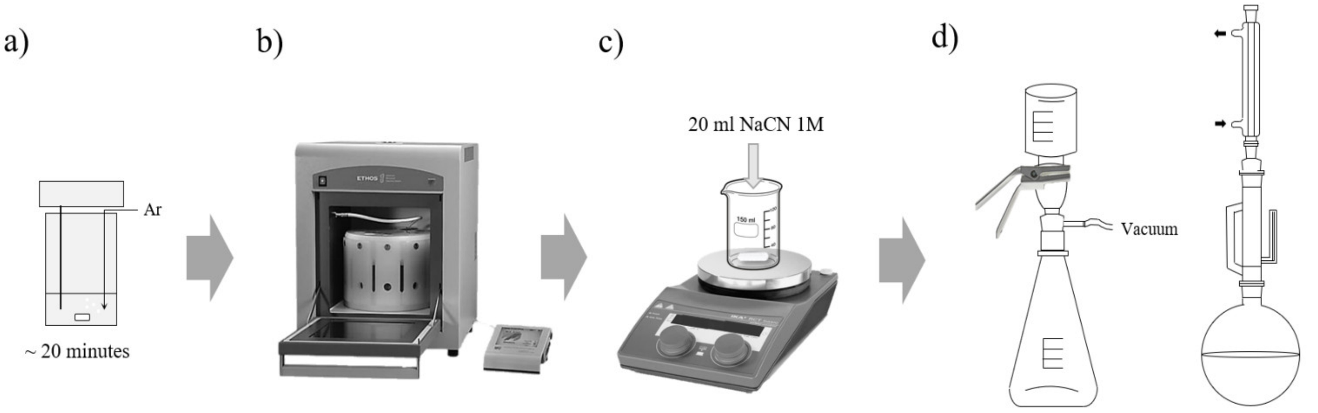

Scheme S1. Preparation route of the three COPs: mixing of reagents and purging with Ar (a), COP synthesis by microwave-assisted hydrothermal method (b), Pd NPs removal by NaCN (c) and final COP extraction (d).

Table S1. Porphyrin and cross-linker molar percentages and ratios of COP-1, COP-2 and COP-3 calculated from combustion chemical analysis data.

Sample	Porphyrin mol%	Cross-linker mol%	Porphyrin: Cross-linker synthesis molar ratio[a]	Porphyrin: Cross-linker real molar ratio[b]
COP-1	38.91	61.09	1 : 2	1 : 1.8
COP-2	46.95	53.05	1 : 1	1 : 1.1
COP-3	49.00	51.00	1 : 1	1 : 1

[a] Synthesis molar ratio corresponds to the quantities of each compound added in the COP synthesis.
[b] Real molar ratio corresponds to the calculated proportion from combustion chemical analysis data of the synthesized COPs presented in Table 1.

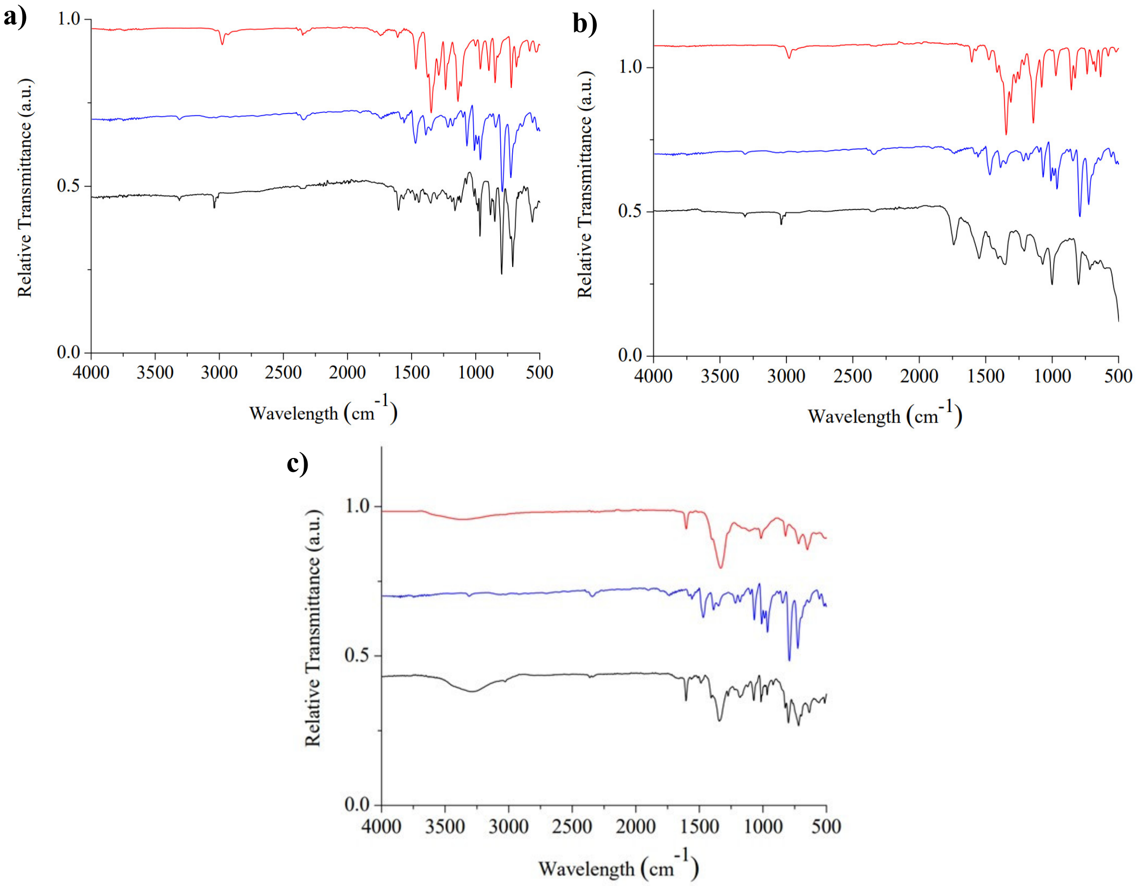

Figure S2. FT-IR spectrums of: a) pyrene-2,7-diboronic acid pinacol ester (red line), 5,10,15,20-(tetra-4-bromophenyl)porphyrin (blue line) and COP-1 (black line); b) 2,2',7,7'-tetrakis(4,4,5,5-tetramethyl-1,3,2-dioxaborolan-2-yl)-9,9'-spirobi[fluorene] (red line), 5,10,15,20-(tetra-4-bromophenyl)porphyrin (blue line) and COP-2 (black line); c) tetra(4-hydroxyboryphenyl)methane (red line), 5,10,15,20-(tetra-4-bromophenyl)porphyrin (blue line) and COP-3 (black line).

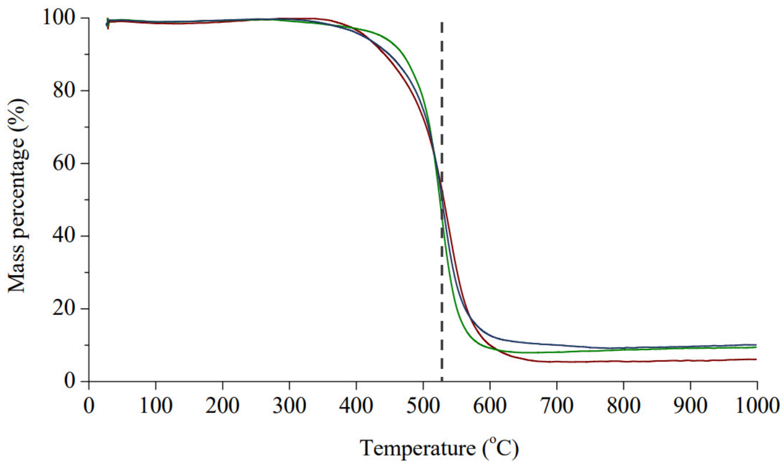

Figure S3. Thermogravimetric analysis performed under air of COP-1 (green line), COP-2 (blue line) and COP-3 (red line).

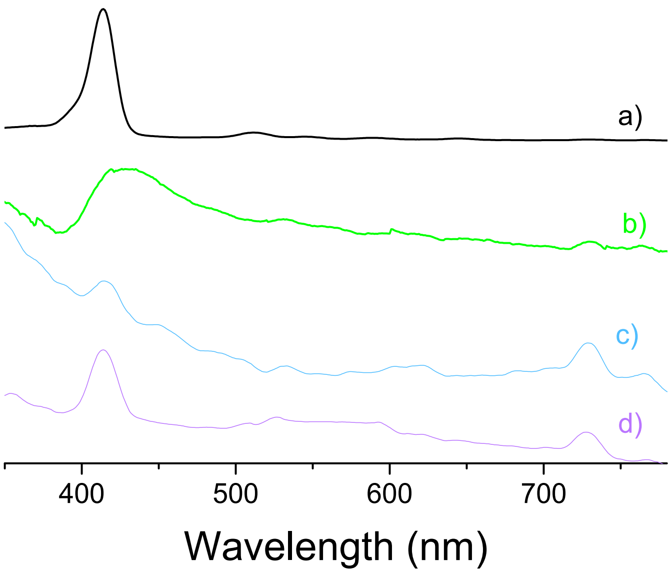

Figure S4. Superposed absorption UV-Vis spectrums in acetonitrile of: a) Porph-Br_4 (black line), b) COP-3 (green line), c) COP-1 (light blue line) and d) COP-2 (violet line).

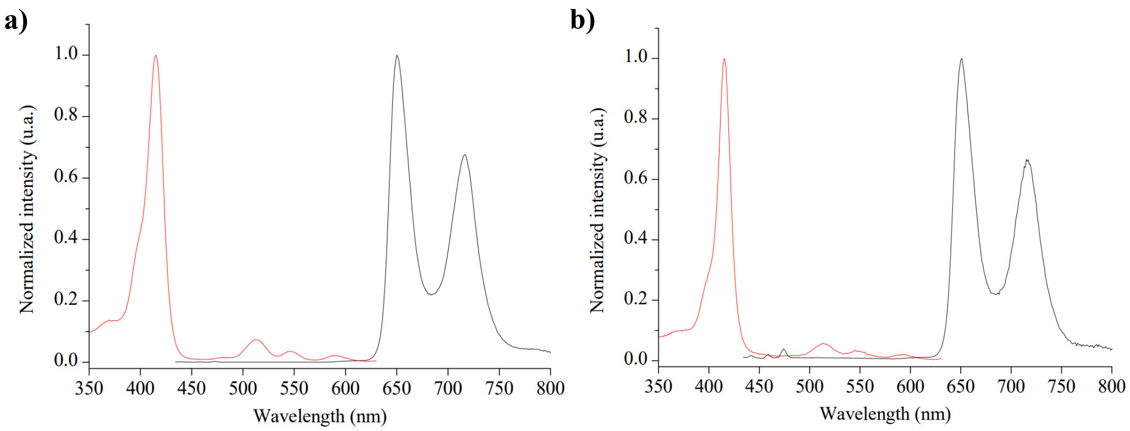

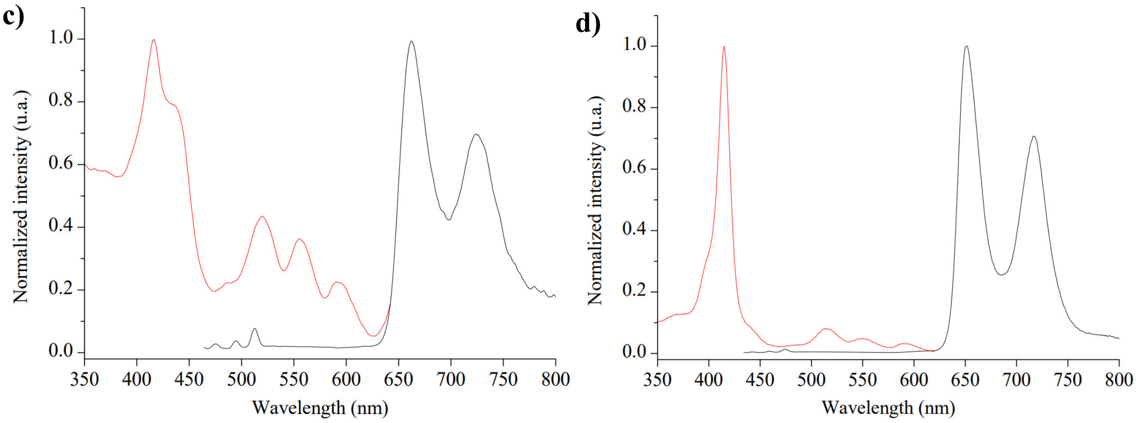

Figure S5. Fluorescence emission spectra (black line, λ_{ex} = 415 nm) and excitation spectra (red line, λ_{em} = 653 nm) recorded in acetonitrile solution of a) Porph-Br$_4$, b) an acetonitrile suspension of COP-1, c) same for COP-2 and d) COP-3.

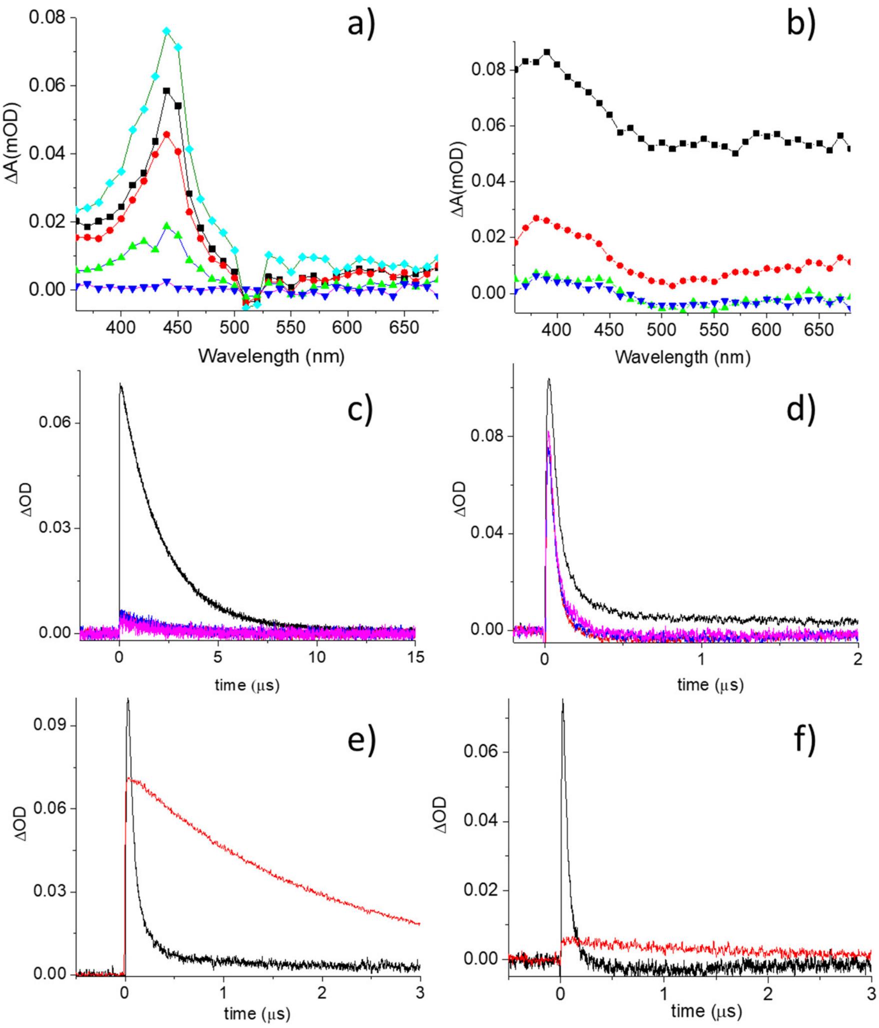

Figure S6. Nanosecond time-resolved transient absorption spectra of very diluted 5,10,15,20-(tetra-4-bromophenyl)porphyrin acetonitrile solution (a) and for a acetonitrile suspension of COP-3 (b). Decay of absorption of the transient excited state of 5,10,15,20-(tetra-4-bromophenyl)porphyrin (c) at 440 (black line), 530 nm (red line), 560 nm (blue line) and 620 nm (pink line) and for COP-3 (d) recorded at 390 nm (black line), 530 nm (red line), 570 nm (blue line) and 630 nm (pink line). (e) Comparison of decays of the transient excited state of starting porphyrin moiety and COP-3 recorded at 440 nm (red line) and 390 nm (black line), respectively. (f) Transient excited state lifetime decay comparison of starting Porph-Br_4 moiety and COP-3 recorded at 440 nm (red line) and 390 nm (black line), respectively.

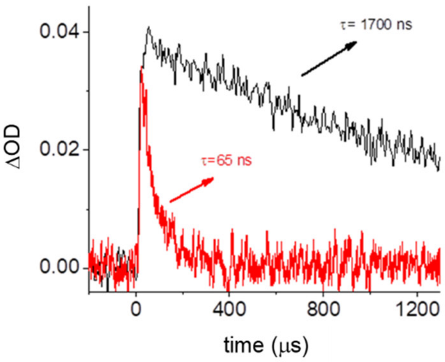

Figure S7. Decay of the transient excited state of Porph-Br_4 before (black line) and after (red line) purging with O_2.

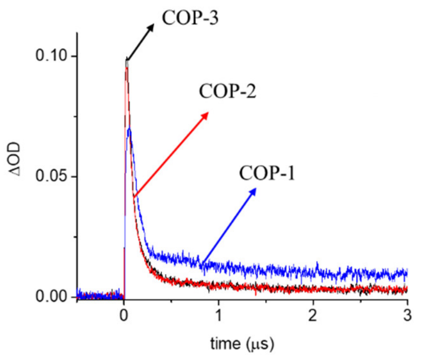

Sample	t_1 (380 nm)	t_2 (380 nm)	Intensity
COP-1	106 ± 1.5 ns	1.5 ± 0.1 μs	0.038
COP-2	69.6 ± 0.2 ns	0.564 ± 0.010 μs	0.096
COP-3	65.3 ± 0.3 ns	0.499 ± 0.008 μs	0.100

Figure S8. Comparison of transient excited state decay at 380 nm of COP-1 (blue line), COP-2 (red line) and COP-3 (black line). **Table S2** summarizes the decay kinetic of three COPs.

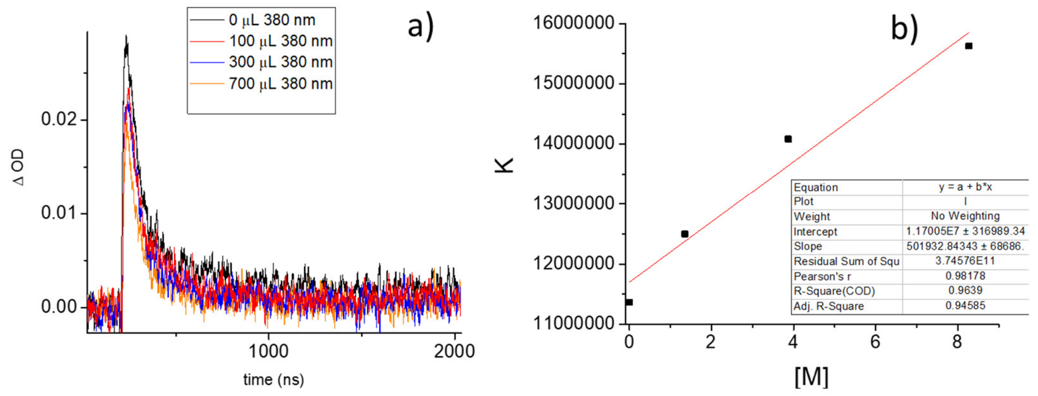

Figure S9. Summarizes quenching data for COP-3 with aliquots of water. (a) Transient absorption excited state decays at 380 nm after adding different amounts of water. (b) Quenching constant calculation plot.

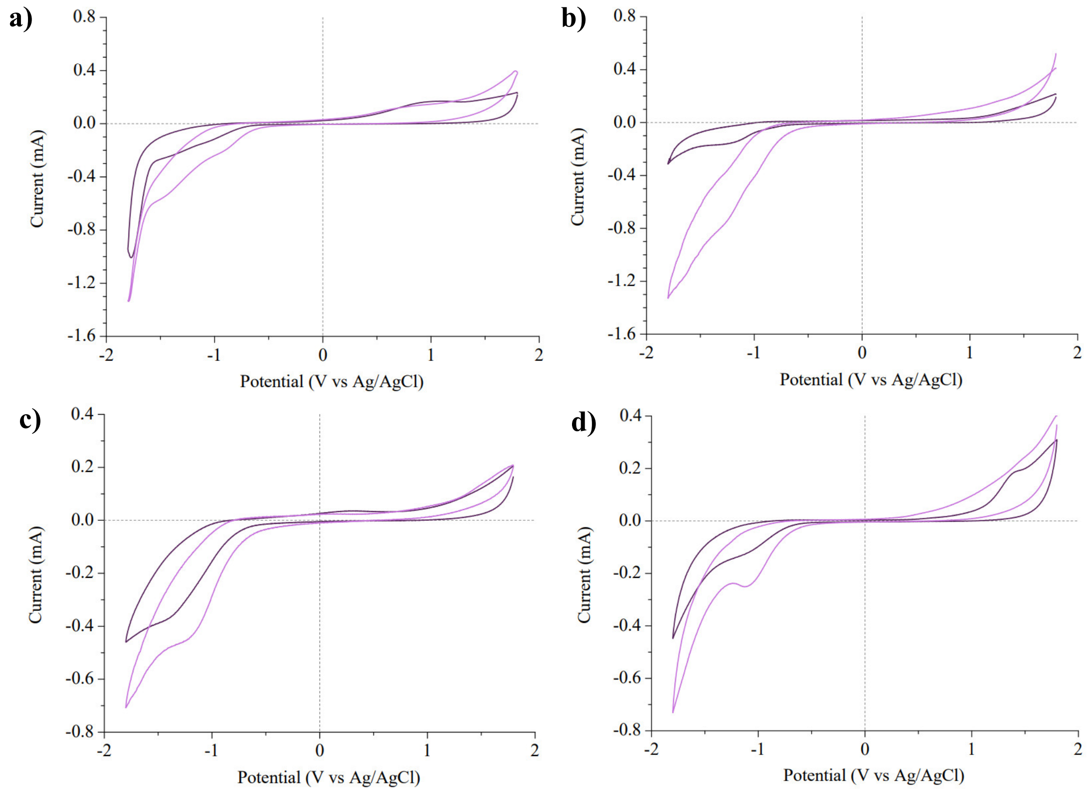

Figure S10. Cyclovoltammetry from 1.8 V to -1.8 V in acetonitrile, in dark (purple line) and under Xe-Hg lamp irradiation with AM 1.5 G light filter (mauve line) of commercial Porph-Br_4 (a), COF-1 (b), COF-2 (c) and COF-3 (d).

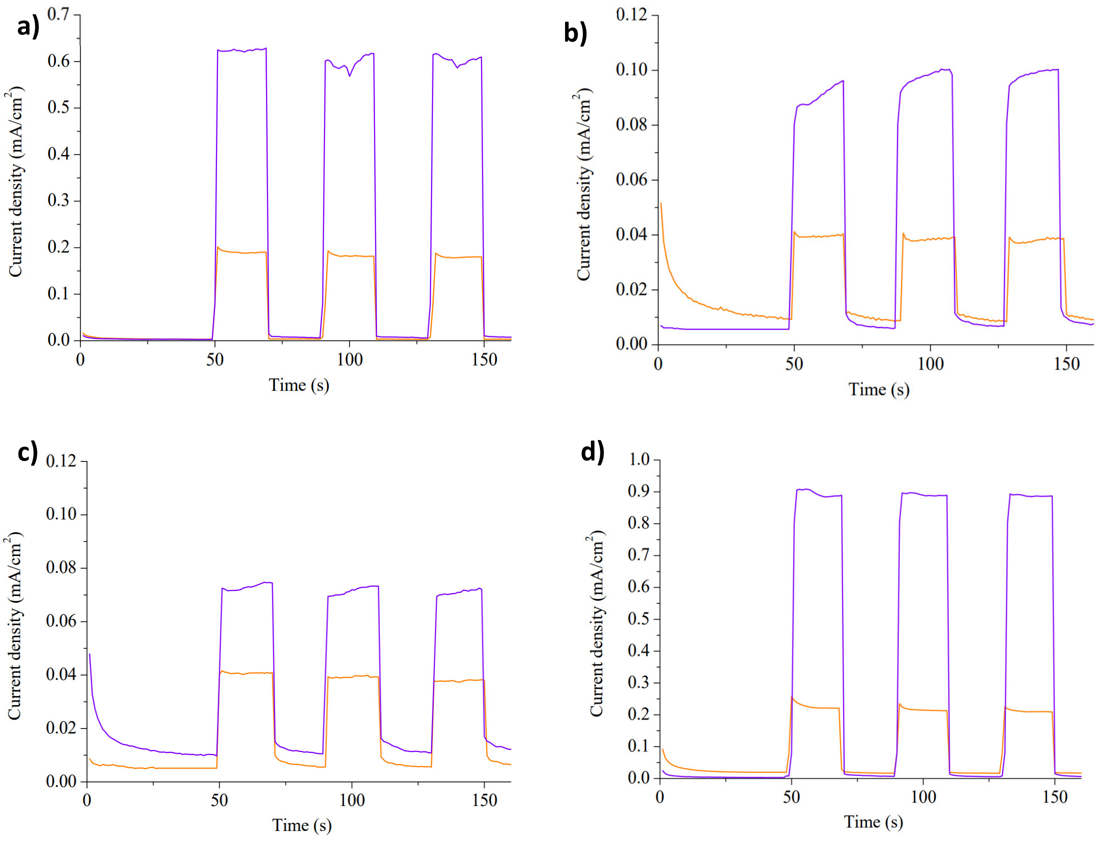

Figure S11. Light on-off photocurrent density response, employing with Pt counter electrode and Ag/AgCl 3 M KCl as reference electrode. Experiments were carried out under Xe-Hg lamp irradiation AM 1.5 G light filter in AcCN with $TBAPF_6$ 0.2 M as electrolyte (orange line) and AcCN with 0.2 M $TBAPF_6$ and 200 μL methanol (mauve line) of: commercial Porph-Br_4 (a), COF-1 (b), COF-2 (c) and COF-3 (d). The applied potential in each experiment was 1.2 V.

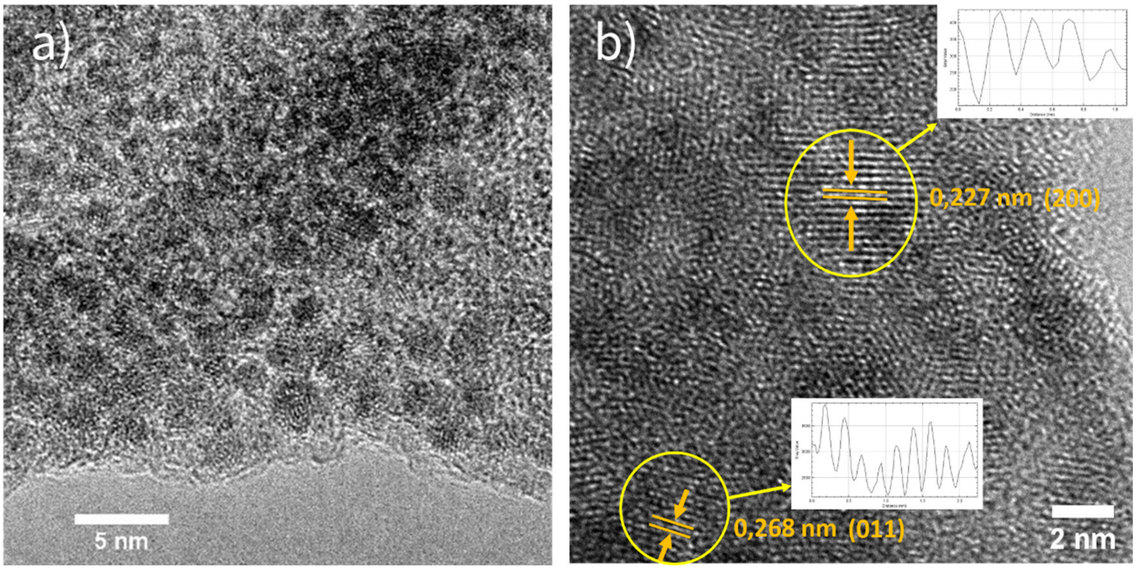

Figure S12. a) TEM image of COP-3 after RuO_2 photo-oxidation and b) HR-TEM of same particle measuring the interplanar distance of the photodeposited RuO_2 nanoparticles that correspond to RuO_2 (011) and (200) crystal phases.

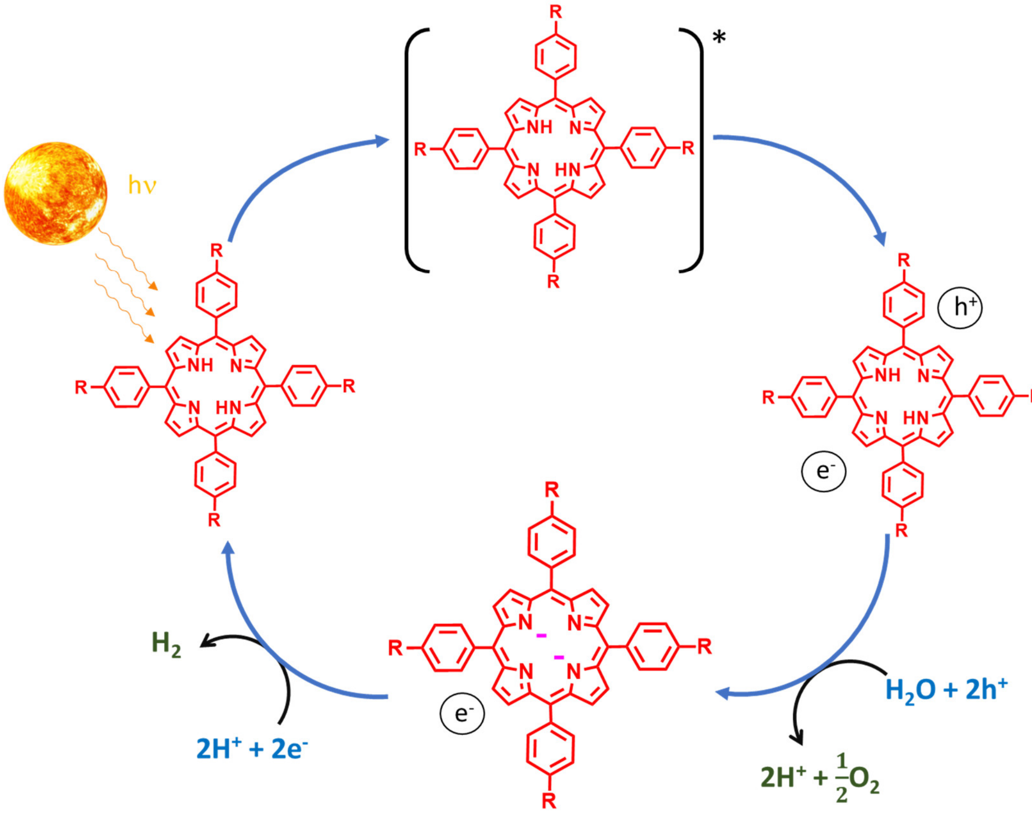

Scheme S*2*. Proposed photocatalytic mechanism for porphyrin-based COPs: (i) excitation of porphyrin moiety upon illumination, (ii) charge separation, (iii) oxidation of water to oxygen and formation of basic form of porphyrin moiety and iv) electron transfer to reduce water to hydrogen.
